# Dynamic predictions of postoperative complications from explainable, uncertainty-aware, and multi-task deep neural networks

**DOI:** 10.1038/s41598-023-27418-5

**Published:** 2023-01-21

**Authors:** Benjamin Shickel, Tyler J. Loftus, Matthew Ruppert, Gilbert R. Upchurch, Tezcan Ozrazgat-Baslanti, Parisa Rashidi, Azra Bihorac

**Affiliations:** 1 Department of Medicine, University of Florida, Gainesville, FL 32611 USA; 2 Department of Surgery, University of Florida, Gainesville, FL 32611 USA; 3 Precision and Intelligent Systems in Medicine (PRISMAp), University of Florida, Gainesville, FL 32611 USA; 4 Department of Biomedical Engineering, University of Florida, Gainesville, FL 32611 USA; 5 Intelligent Health Lab (i-Heal), University of Florida, Gainesville, FL 32611 USA; 6 Intelligent Critical Care Center (IC3), University of Florida, Gainesville, FL 32611 USA

**Keywords:** Computational science, Surgery

## Abstract

Accurate prediction of postoperative complications can inform shared decisions regarding prognosis, preoperative risk-reduction, and postoperative resource use. We hypothesized that multi-task deep learning models would outperform conventional machine learning models in predicting postoperative complications, and that integrating high-resolution intraoperative physiological time series would result in more granular and personalized health representations that would improve prognostication compared to preoperative predictions. In a longitudinal cohort study of 56,242 patients undergoing 67,481 inpatient surgical procedures at a university medical center, we compared deep learning models with random forests and XGBoost for predicting nine common postoperative complications using preoperative, intraoperative, and perioperative patient data. Our study indicated several significant results across experimental settings that suggest the utility of deep learning for capturing more precise representations of patient health for augmented surgical decision support. Multi-task learning improved efficiency by reducing computational resources without compromising predictive performance. Integrated gradients interpretability mechanisms identified potentially modifiable risk factors for each complication. Monte Carlo dropout methods provided a quantitative measure of prediction uncertainty that has the potential to enhance clinical trust. Multi-task learning, interpretability mechanisms, and uncertainty metrics demonstrated potential to facilitate effective clinical implementation.

## Introduction

In the United States, more than 15 million major, inpatient surgeries are performed each year^1^. Complications occur in up to 32%; major complications decrease quality of life and increase health care costs by as much as $11,000^2,3^. Accurate, personalized predictions of postoperative complications can inform shared decisions between patients and surgeons regarding prognosis, the appropriateness of surgery, prehabilitation strategies targeting modifiable risk factors (e.g., smoking cessation), and postoperative resource use (e.g., triage to intensive care or general wards), suggesting opportunities to augment clinical risk prediction with objective, machine learning-enabled decision-support.

Most existing perioperative predictive analytic decision-support tools are hindered by suboptimal performance, time constraints imposed by manual data entry requirements, and lack of intraoperative data and clinical workflow integration^4–9^. These challenges are theoretically mitigated by automated deep learning models that capture latent, nonlinear data structure and relationships among raw feature representations in large datasets^10^, now widely available in electronic health records (EHRs)^11^. Despite these potential advantages^12–20^, deep learning using the full spectrum of preoperative and intraoperative, patient-specific EHR data to predict postoperative complications has not been previously reported. Recognition that deep learning models with high overall accuracy are nevertheless capable of egregious errors, along with their lack of interpretability, have invited skepticism regarding the clinical application of deep learning-enabled decision-support; model interpretability and uncertainty-awareness mechanisms have the potential to improve clinical applicability, but their efficacy remains unclear.

Using a longitudinal cohort of 56,242 patients who underwent 67,481 inpatient surgeries, we test the hypotheses that deep learning models would outperform random forest and XGBoost baseline models in predicting postoperative complications using both preoperative and intraoperative physiological time series data. We also explore the utility of multi-task learning^21,22^ by training a single deep learning model on several postoperative complications simultaneously to improve model efficiency, integrated gradients to promote model interpretability, and uncertainty metrics that represent variance across predictions.

## Results

### Participant baseline characteristics and outcomes

Cohort characteristics are summarized in Table 1 and detailed cohort statistics are presented in Supplementary Tables S1–S4. The overall study population had mean age 56 years and 50% were female. In the validation cohort of 20,293 surgical procedures, the incidence of complications was: 33.3% prolonged ICU stay (for 48 h or more), 7.8% prolonged mechanical ventilation, 20.2% neurological complications, 16.9% acute kidney injury, 16.3% cardiovascular complications, 5.4% venous thromboembolism, 21.4% wound complications, 8.7% sepsis, and 1.6% in-hospital mortality. The distribution of complications was similar between development and validation cohorts.

**Table 1:** Summary of development and validation cohorts.

	Development cohort (6/1/2014–11/26/2018)	Validation cohort (11/27/2018–9/20/2020)
Patients, n	38,621	17,621
Hospital encounters, n	47,188	20,293
Age, years, median (25th, 75th)	59.0 (45.0, 69.0)	61.0 (47.0, 71.0)
Length of stay, days, median (25th, 75th)	4.1 (2.2, 7.9)	4.3 (2.2, 8.4)
Length of surgery, hours, median (25th, 75th)	3.1 (2.2, 4.6)	3.2 (2.3, 4.7)
Emergent admission, n (%)	16,706 (35.4%)	7491 (36.9%)
Charlson comorbidity index, median (25th, 75th)	4.0 (2.0, 6.0)	4.0 (2.0, 6.0)
**Sex, n (%)**		
Female	23,716 (50.3%)	10,005 (49.3%)
Male	23,472 (49.7%)	10,288 (50.7%)
**Race, n (%)**		
White	37,047 (78.5%)	15,942 (78.6%)
African American	6562 (13.9%)	2759 (13.6%)
Other/Unknown	3579 (7.6%)	1592 (7.8%)
**Admission type, n (%)**		
Medicine	20,893 (44.3%)	8277 (40.8%)
Surgery	17,899 (37.9%)	7806 (38.5%)
Other	8396 (17.8%)	4210 (20.7%)
**Postoperative complications, n (%)**		
Prolonged ICU Stay (> 2 Days)	12,980 (27.5%)	6765 (33.3%)
Prolonged mechanical ventilation (> 2 Days)	3512 (7.4%)	1574 (7.8%)
Wound complications	6782 (14.4%)	4347 (21.4%)
Neurological complications	7273 (15.4%)	4107 (20.2%)
Cardiovascular complications	5655 (12.0%)	3301 (16.3%)
Sepsis	3445 (7.3%)	1775 (8.7%)
Acute kidney injury	6894 (14.6%)	3438 (16.9%)
Venous thromboembolism	2008 (4.3%)	1101 (5.4%)
In-hospital mortality	788 (1.7%)	321 (1.6%)

### Multi-task learning improved efficiency without compromising predictive performance

**Figure 1:**
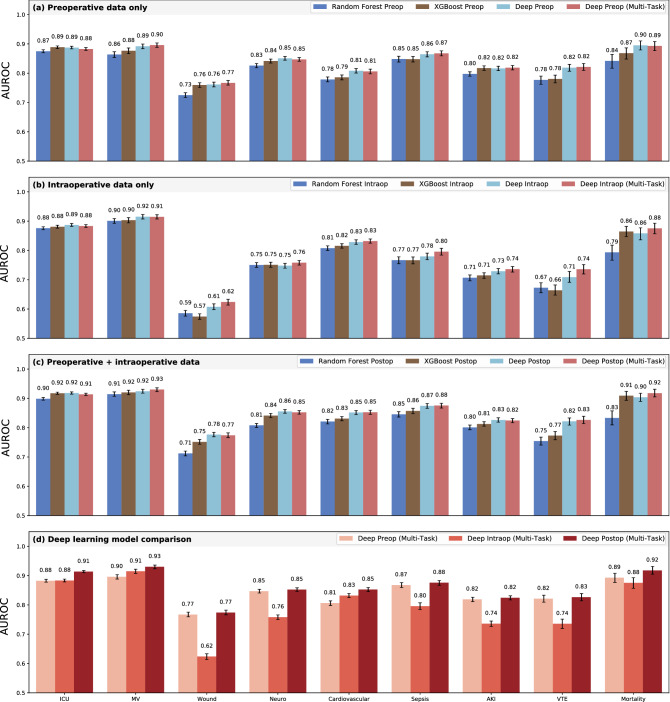
Classification accuracy compared with baseline models. Shown are area under the receiver operating characteristic curve (AUROC) results for random forest and XGBoost models, individual deep learning models independently trained on each outcome, and a combined multi-task jointly trained on all outcomes, using only preoperative features (**a**), only intraoperative features (**b**), and both preoperative and intraoperative features (**c**). A comparison of multi-task deep learning results at three stages of prediction is shown in (**d**).

For deep learning models trained on preoperative data alone, there were no significant differences between multi-task outcome-specific models. For models trained on intraoperative time series alone, the multi-task model yielded significantly higher AUROC for sepsis (0.80 [95% confidence interval 0.78–0.81] vs. 0.78 [0.77–0.79]) and venous thromboembolism (0.74 [0.72–0.75] vs. 0.71 [0.69–0.73]). Using all available preoperative and intraoperative data, the multi-task postoperative model yielded somewhat higher AUROC for prolonged mechanical ventilation, sepsis, venous thromboembolism, and in-hospital mortality, and lower AUROC for prolonged ICU stay, wound complications, neurological complications, and acute kidney injury, though the differences were not statistically significant. A comprehensive AUROC comparison between individual models and multi-task learning is shown in Fig. 1a–c. Given that multi-task models had marginally stronger performance and have a reduced computational requirements and training times compared with nine individual models, the multi-task approach is used henceforth as our deep learning-based postoperative model, unless stated otherwise. Full results are shown in Supplementary Table S5.

### Deep learning outperformed random forest and XGBoost baseline models

Deep learning and baseline models (random forest, XGBoost) used the same feature sets with one exception: due to the nature of sequential deep learning methods, our deep intraoperative models processed the entire physiological time series minute-by-minute, whereas the baseline intraoperative and postoperative models required extraction of summary statistics. A full list of random forest and XGBoost time series features is described in Supplementary Table S6. A full comparison among all models, performance metrics, and complication outcomes is described in Supplementary Methods and Supplementary Table S5.

#### Preoperative models

The deep multi-task model trained only on static, preoperative descriptors yielded higher AUROC compared with random forest models for all nine outcomes, with significant performance increases for prolonged mechanical ventilation (0.90 [0.89–0.90] vs. 0.86 [0.85–0.87]), wound complications (0.77 [0.76–0.78] vs. 0.73 [0.72–0.73]), neurological complications (0.85 [0.84–0.85] vs. 0.83 [0.82–0.83]), cardiovascular complications (0.81 [0.80–0.81] vs. 0.78 [0.77–0.79]), acute kidney injury (0.82 [0.81–0.83] vs. 0.80 [0.79–0.80]), venous thromboembolism (0.82 [0.81–0.83] vs. 0.78 [0.76–0.79]), and in-hospital mortality (0.89 [0.88–0.91] vs. 0.84 [0.82–0.86]).

Compared with XGBoost, deep learning models yielded superior AUROC for all outcomes except for prolonged ICU stay and acute kidney injury, in which the two models performed equivalently. Deep learning yielded significant AUROC improvements for cardiovascular complications (0.81 [0.80–0.82] vs. 0.79 [0.78–0.79]) and venous thromboembolism (0.82 [0.81–0.83] vs. 0.78 [0.77–0.79]).

#### Intraoperative models

Using intraoperative time series input data alone, multi-task deep learning yielded higher AUROC compared with random forests for all complications except prolonged ICU stay, for which AUROC was equivalent. Significant AUROC improvements were yielded for wound complications (0.62 [0.61–0.63] vs. 0.59 [0.58–0.60]), acute kidney injury (0.74 [0.73–0.74] vs. 0.71 [0.70–0.72]), venous thromboembolism (0.74 [0.72–0.75] vs. 0.67 [0.66–0.69]), and in-hospital mortality (0.88 [0.86–0.89] vs. 0.79 [0.77–0.82]).

Compared with XGBoost, deep learning resulted in superior AUROC for all nine outcomes, with significant improvements for wound complications (0.62 [0.61–0.63] vs. 0.57 [0.56–0.58]), acute kidney injury (0.74 [0.73–0.74] vs. 0.71 [0.70–0.72]), and venous thromboembolism (0.74 [0.72–0.75] vs. 0.66 [0.65–0.68]).

#### Postoperative models

The deep postoperative multi-task model trained on all available data yielded significant higher AUROC compared with a random forest model for eight of nine complications, including prolonged ICU stay (0.91 [0.91–0.92] vs. 0.90 [0.89–0.90]), wound complications (0.77 [0.77–0.78] vs. 0.71 [0.70–0.72]), neurological complications (0.85 [0.85–0.86] vs. 0.81 [0.80–0.81]), cardiovascular complications (0.85 [0.85–0.86] vs. 0.82 [0.81–0.83]), sepsis (0.88 [0.87–0.88] vs. 0.85 [0.84–0.85]), acute kidney injury (0.82 [0.82–0.83] vs. 0.80 [0.79–0.81]), venous thromboembolism (0.83 [0.81–0.84] vs. 0.75 [0.74–077]), and in-hospital mortality (0.92 [0.91–0.93] vs. 0.83 [0.81–0.86]). The deep multi-task model yielded somewhat higher AUROC for prolonged mechanical ventilation, but the difference was not statistically significant.

Compared with XGBoost, deep learning resulted in superior AUROC for all outcomes except for prolonged ICU stay, in which the performance was equivalent. Deep learning yielded significant AUROC improvements for wound complications (0.78 [0.77–0.78] vs. 0.75 [0.74–0.76]), cardiovascular complications (0.85 [0.85–0.86] vs. 0.83 [0.82–0.84]), and venous thromboembolism (0.83 [0.81–0.84] vs. 0.77 [0.76–0.79]).

A full AUROC comparison between deep learning, random forest, and XGBoost models is shown in Fig. 1a–c and Supplementary Table S5.

### Deep postoperative models outperformed deep preoperative models

Compared with deep preoperative models, deep postoperative models had significantly higher AUROC for prolonged ICU stay (0.91 [95% confidence interval 0.91–0.92] vs. 0.88 [0.88–0.89]), prolonged mechanical ventilation (0.93 [0.92–0.94] vs. 0.90 [0.89–0.90]), and cardiovascular complications (0.85 [0.85–0.86] vs. 0.81 [0.80–0.81]). A full comparison is shown in Fig. 1d. Using deep multi-task preoperative predictions as a benchmark, the deep multi-task postoperative models made significant overall reclassification improvements for prolonged ICU stay (overall, correctly reclassified 3.7% of all surgical encounters, *p* < 0.01), prolonged mechanical ventilation (overall, correctly reclassified 4.8%, *p* < 0.01), and cardiovascular complications (overall, correctly reclassified 0.3%, *p* < 0.01). There were no statistically significant declines in reclassification. In some cases, deep models for individual complications yielded better net reclassification indices than multi-task models, including wound complications (− 1.7% vs. − 2.9%, *p* < 0.01) and cardiovascular complications (2.8% vs. 0.3%, *p* < 0.01). Full net reclassification results are shown in Supplementary Table S7. Detailed statistics for absolute and relative risk between preoperative and postoperative models are shown in Supplementary Table S8, and analyses of risk group transitions are shown in Supplementary Tables S9 and S10.

### Model uncertainty

We applied the method of Monte Carlo dropout to derive measures of prediction uncertainty, representing variance across predictions, for each of our deep learning models. Uncertainty results for each prediction phase and training procedure are shown in Table 2, where uncertainty is expressed as prediction variance over 100 stochastic trials using dropout at inference time. Interestingly, models trained only using intraoperative data resulted in the lowest uncertainty for each postoperative complication. Within each outcome and prediction phase, individual models yielded lower predictive uncertainty compared with multi-task model counterparts. Using the models with the least uncertain training scheme for each outcome and prediction phase, postoperative predictions were less uncertain than preoperative predictions for prolonged mechanical ventilation, wound complications, cardiovascular complications, and in-hospital mortality; postoperative uncertainty was higher for the remaining five complications.

**Table 2:** Deep model uncertainty metrics aggregated over 100 Monte Carlo dropout iterations.

Outcome	Prediction point	Model type	Mean uncertainty (variance × 10^3^)	Mean AUROC
Prolonged ICU stay	Preop	Individual	3.471	0.887
		Multi-task	5.907	0.883
	Intraop	Individual	1.178	0.887
		Multi-task	1.401	0.884
	Postop	Individual	3.675	0.919
		Multi-task	4.785	0.914
Prolonged MV	Preop	Individual	2.695	0.892
		Multi-task	4.040	0.896
	Intraop	Individual	0.851	0.915
		Multi-task	1.274	0.916
	Postop	Individual	2.345	0.925
		Multi-task	3.122	0.931
Wound	Preop	Individual	3.058	0.761
		Multi-task	6.191	0.767
	Intraop	Individual	0.553	0.608
		Multi-task	1.077	0.624
	Postop	Individual	2.884	0.777
		Multi-task	5.363	0.774
Neuro	Preop	Individual	2.194	0.851
		Multi-task	4.856	0.847
	Intraop	Individual	1.553	0.748
		Multi-task	1.326	0.758
	Postop	Individual	2.723	0.855
		Multi-task	4.270	0.852
Cardiovascular	Preop	Individual	2.331	0.809
		Multi-task	3.657	0.806
	Intraop	Individual	0.967	0.829
		Multi-task	1.177	0.833
	Postop	Individual	2.209	0.852
		Multi-task	2.802	0.853
Sepsis	Preop	Individual	1.751	0.864
		Multi-task	3.922	0.868
	Intraop	Individual	1.533	0.780
		Multi-task	1.643	0.796
	Postop	Individual	1.885	0.875
		Multi-task	4.030	0.876
AKI	Preop	Individual	2.337	0.816
		Multi-task	4.541	0.819
	Intraop	Individual	0.955	0.730
		Multi-task	1.200	0.737
	Postop	Individual	3.146	0.826
		Multi-task	3.827	0.824
Venous thromboembolism	Preop	Individual	1.781	0.819
		Multi-task	4.857	0.821
	Intraop	Individual	0.677	0.709
		Multi-task	1.173	0.735
	Postop	Individual	2.688	0.821
		Multi-task	5.000	0.827
In-hospital mortality	Preop	Individual	1.853	0.895
		Multi-task	3.675	0.893
	Intraop	Individual	0.727	0.858
		Multi-task	1.520	0.876
	Postop	Individual	1.820	0.903
		Multi-task	3.452	0.918

### Model interpretability

We applied integrated gradients to our multi-task deep learning postoperative prediction model. The top 10 features per complication outcome for every sample in the validation cohort are shown with corresponding attribution scores in Table 3. Importance distribution among the top 10 features per complication are visualized in Supplementary Fig. S1, and distributions of feature importance values grouped by input and feature type are visualized in Supplementary Figs. S2 and S3. The important feature lists, as described in subsequent sections, are consistent with medical knowledge, experience, and evidence, establishing an important element in gaining the trust of patients and clinicians^23^.

**Table 3:** The 10 most influential features using integrated gradients aggregated over validation cohort.

Prolonged ICU stay	Prolonged mechanical ventilation	Wound complications	Neurological complications	Cardiovascular complications	Sepsis	Acute kidney injury	Venous thromboembolism	In-hospital mortality
Peak inspiratory pressure (0.068)	Fraction of inspired oxygen (0.067)	Primary procedure (0.066)	Primary procedure (0.037)	Systolic blood pressure (0.068)	Heart rate (0.045)	Creatinine, serum (0.034)	Primary procedure (0.044)	Primary procedure (0.039)
Heart rate (0.063)	Peak inspiratory pressure (0.053)	Surgeon specialty (0.043)	Surgery type (0.035)	Peak inspiratory pressure (0.064)	Primary procedure (0.039)	Primary procedure (0.032)	Heart rate (0.036)	Minimum alveolar concentration (0.035)
Blood oxygen saturation (0.062)	Heart rate (0.053)	Attending surgeon (0.037)	Blood oxygen saturation (0.034)	Blood oxygen saturation (0.061)	Surgeon specialty (0.028)	Surgeon specialty (0.032)	Prothrombin time, serum (0.033)	Blood oxygen saturation (0.032)
Systolic blood pressure (0.048)	Blood oxygen saturation (0.044)	Surgery type (0.033)	Peak inspiratory pressure (0.033)	Heart rate (0.056)	Scheduled surgery room (0.025)	Attending surgeon (0.030)	Peak inspiratory pressure (0.026)	Peak inspiratory pressure (0.028)
Diastolic blood pressure (0.040)	Primary procedure (0.029)	Scheduled surgery room (0.026)	Erythrocytes, urine (0.032)	Diastolic blood pressure (0.045)	Blood oxygen saturation (0.024)	Peak inspiratory pressure (0.030)	Surgeon specialty (0.025)	Scheduled surgery room (0.026)
Primary procedure (0.031)	Respiratory rate (0.029)	ZIP code (0.025)	Minimum alveolar concentration (0.030)	Minimum alveolar concentration (0.033)	ZIP code (0.024)	Surgery type (0.028)	Blood oxygen saturation (0.025)	Erythrocytes, urine (0.026)
Fraction of inspired oxygen (0.030)	Scheduled surgery room (0.028)	Heart rate (0.025)	Scheduled surgery room (0.026)	Core temperature (0.030)	Surgery type (0.023)	Blood oxygen saturation (0.026)	Fraction of inspired oxygen (0.025)	Diastolic blood pressure (0.026)
Surgery duration (0.027)	Tidal volume (0.027)	Surgery duration (0.024)	Diastolic blood pressure (0.026)	Respiratory rate (0.021)	Erythrocytes, urine (0.023)	Heart rate (0.022)	Erythrocyte distribution width (0.024)	Fraction of inspired oxygen (0.022)
Mean arterial pressure (0.025)	Systolic blood pressure (0.023)	Albumin, serum (0.022)	Heart rate (0.025)	Primary procedure (0.021)	Peak inspiratory pressure (0.020)	Surgery duration (0.021)	Attending surgeon (0.023)	ZIP code (0.022)
Surgery type (0.023)	ZIP code (0.022)	Platelet mean volume (0.021)	Systolic blood pressure (0.024)	Surgery duration (0.020)	Attending surgeon (0.019)	Urea nitrogen, serum (0.020)	ZIP code (0.023)	Surgery type (0.021)

#### Prolonged ICU stay

The most important feature was peak inspiratory pressure; the presence of such a value indicates the performance of mechanical ventilation, and higher values could indicate intrinsic lung disease, proximal airway or breathing tube narrowing or obstruction, or the transmission of increased intra-abdominal pressure, each of which suggest greater illness severity. The next two most important features were heart rate and blood oxygen saturation, both of which are major determinants of cardiac output and oxygen delivery.

#### Prolonged mechanical ventilation

**Figure 2:**
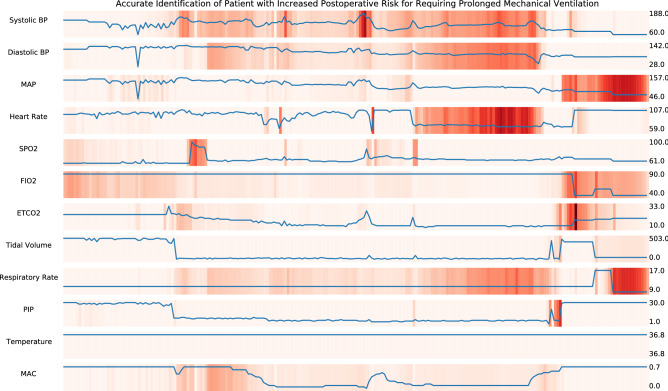
Temporal integrated gradients feature attributions for example patient experiencing prolonged mechanical ventilation. The multi-task deep learning model correctly predicted elevated risk of prolonged mechanical ventilation after integrating multivariate intraoperative time series. Physiological time series labeled by variable (left) and value range (right). Implementation of integrated gradients highlighted physiological patterns important for updated risk prediction, including a rapid increase in heart rate and ETCO2, fluctuations in PIP, and changes in SPO2. ETCO2, end-tidal carbon dioxide; PIP, peak inspiratory pressure; SPO2, blood oxygen saturation.

Peak inspiratory pressure and heart rate were again top features, along with fraction of inspired oxygen, the number one feature. This result is consistent with prior observations that most etiologies of hypoxemia improve with increasing fraction of inspired oxygen, apart from right-to-left shunt, which is often accompanied by another pathophysiologic process that is responsive to higher fraction of inspired oxygen. Temporal feature attributions for physiological intraoperative time series from an example patient requiring prolonged mechanical ventilation are shown in Fig. 2, with another example for a patient developing cardiovascular complications shown in Supplementary Fig. S4.

#### Wound complications

The major factors affecting wound complications (i.e., infection, dehiscence, and non-healing) are the type of surgery and its associated degree of wound contamination^24,25^. These factors are aligned with the top five important features for wound complication prediction: primary procedure, surgeon specialty, attending surgeon, surgery type, and scheduled surgery room. Although body mass index is unexpectedly missing from the top 10 feature list, several other factors relate to known risk factors for wound complications, including malnutrition, long duration of surgery, blood loss, and anemia.

#### Neurological complications

Similar to wound complications, neurological complications are primarily a function of type of surgery; neurosurgical procedures typically involve pre-existing neurological pathology and confer above-average risk for postoperative neurological pathology relative to other types of surgery. Accordingly, primary procedure and surgery type were the top two important features in predicting neurological complications.

#### Cardiovascular complications

Cardiovascular complications may be caused by or lead to cardiac and respiratory pathophysiology, primarily measured by cardiac and respiratory vital signs and mechanical ventilator measurements^26^. Consistent with these phenomena, the top five important features for cardiovascular complications were systolic blood pressure, peak inspiratory pressure, blood oxygen saturation, heart rate, and diastolic blood pressure.

#### Sepsis

Important features for sepsis were similar to those of wound complications, with the exception of heart rate, which was the most important feature for sepsis. One might expect that fever, leukocytosis, and hypotension would be important features in predicting sepsis, but it is possible that these elements would occur later after surgery when sepsis was developing as a postoperative complication, and they can also represent sterile postoperative inflammation from tissue damage without infection. Heart rate variability, which would be learned from intraoperative time series heart rate values, is well established as a strong predictor of sepsis and associated adverse outcomes^27,28^.

#### Acute kidney injury

Serum creatinine is the primary method for measuring kidney function among hospitalized patients and tends to be more reliable than volume of urine output, which is difficult to record accurately in the absence of an indwelling bladder catheter. Accordingly, the number one important feature in predicting acute kidney injury was serum creatinine. Several other important features represented kidney perfusion or red blood cell production, which is affected by the endogenous renal hormone erythropoietin.

#### Venous thromboembolism

Major risk factors for venous thromboembolism are encompassed by Virchow’s triad of vessel injury, altered blood flow, and hypercoagulability^29^. These elements are represented in two of the top three important features for predicting venous thromboembolism (i.e., primary procedure and serum prothrombin time), as well as several other variables in the top 10 feature list.

## Discussion

In predicting postoperative complications among adult patients undergoing major, inpatient surgery, deep neural networks outperformed random forest and XGBoost classifiers, exhibiting strongest performance when leveraging the full spectrum of preoperative and intraoperative EHR data. Intraoperative physiological time-series had meaningful associations with postoperative patient outcomes, suggesting that prediction models augmented with intraoperative data may have utility for routine clinical tasks such as sharing prognostic information with patients and caregivers and making clinical management decisions regarding triage destination and resource use after surgery. Deep models maintained high performance using efficient multi-task methods predicting nine complications simultaneously, rather than predicting individual complications with separate models that require extra training time. Uncertainty metrics revealed that variance across model predictions is lowest when using intraoperative data alone, consistent with the perspective that many preoperative EHR predictor variables represent clinician decision-making (e.g., the lack of preoperative bilirubin values indicates a decision to forego hepatic function testing) rather than pure physiology, and therefore introduce greater variance in predictions. Finally, applying integrated gradients interpretability methods elucidated feature importance patterns that were biologically plausible and consistent with medical knowledge, experience, and evidence, harboring the potential to gain trust from patients and clinicians^23^.

Previous studies have established that for many clinical prediction tasks, deep neural networks outperform other methods, such as logistic regression classifiers^30,31^. Parametric regression equations often fail to accurately represent complex, non-linear associations among input variables, limiting their predictive performance. More than 30 years ago, Schwartz et al.^32^ suggested that human disease is too broad and complex to be accurately represented by rule-based algorithms, and that machine learning models obviate this limitation by learning from data. In our study, deep learning also outperformed random forest and XGBoost models, likely because the deep models capitalized on the availability and granularities of intraoperative time series data. As EHR data volumes expand, deep learning healthcare applications gain greater potential for clinical application^33^. However, this will require integration with real-time clinical workflow. Therefore, it seems prudent to design models that make updated predictions as EHR data become available. We sought to achieve this objective by using recurrent neural networks that can update their predictions when new data becomes available. Our results suggest that these models would perform well in prospective clinical settings.

Multi-task methods did not yield predictive performance advantages in our study, but they have yielded performance advantages in previous studies. Multi-task learning can improve model generalizability by penalizing the exploration of certain regions of the available function space, thus reducing overfitting from the false assumption that data noise is sparse or absent. This has been demostrated by Si and Roberts^34^ in applying CNN multi-task learning to word embeddings in MIMIC-III clinical notes data, demonstrating that multi-task learning models outperformed single-task models in predicting mortality within 1, 3, 5, and 20 different timeframes. In addition, multi-task learning can act as a regulizer for learning classifiers from a finite set of examples by penalizing complexity in a loss function, as demonstrated by Harutyunyan et al.^21^ in predicting mortality and physiological decompensation among ICU patients in the publicly available MIMIC-III database^35^. However, multi-task learning was not advantageous for phenotyping acute care conditions; the authors postulated that this occurred because phenotyping is multi-task by nature, i.e., already benefits from regularization across phenotypes. This may not hold true for rare, complex phenotypes, for which multi-task learning can reduce neural network sensitivity to hyperparameter settings (i.e., parameters that are set before learning begins), as demonstrated by Ding et al.^36^ Properly applied, multi-task learning can improve model generalizability and classification in deep learning clinical prediction models, optimizing performance and usability across diverse settings and datasets, with the added advantage of reduced model training times relative to training multiple individual models.

One barrier to clinical adoption of deep learning clinical prediction models is difficulty interpreting outputs. Patients, caregivers, and clinicians may be more willing to incorporate model predictions in shared decision-making processes if they understand how and why a prediction was made and believe that the prediction is consistent with medical knowledge and evidence. Integrated gradients techniques attempt to explain predictions made by deep learning models, usually by feeding perturbed inputs to the model, evaluating effects on outputs, and using this information to quantify and convey feature importance. Sayres et al.^37^ used integrated gradients to identify retinal image regions contributing to deep learning-based diabetic retinopathy diagnoses, which was associated with improved ophthalmologist diagnostic accuracy and confidence. These methods have the potential to facilitate clinical adoption of deep learning prediction models by allowing patients, caregivers, and clinicians to understand how and why an output was produced. Finally, demonstrating low variance across predictions with uncertainty metrics could assuage well-founded patient and clinician fears that an individual model output represents a rare but egregious prediction error, for which deep learning models are infamous.

This study was limited by its single-institution, retrospective design. Although multi-task functions may reduce overfitting, the use of data from a single institution limits generalizability. Our models have not been tested using prospective, real-time data, which may present data pre-processing challenges. Future research should seek prospective, multi-center validation of these findings. While we describe our data processing, modeling, and experimental approach in suitable depth to allow individual reproducibility on other private datasets, our results may not result in more broadly generalizable findings. In the current data sharing climate, comprehensive external validation will be difficult to perform until cloud sharing of standardized EHR data or federated learning are achieved at scale^38^. Our deep learning models also use a relatively straightforward implementation of multi-task learning; future work will explore the impact of more complex formulations of cross-task knowledge transfer. Finally, it remains unknown how the predictions generated by models presented herein would affect shared decision-making processes and patient outcomes.

In summary, deep learning yielded greater discrimination than random forest and XGBoost models for predicting complications after major, inpatient surgery. Uncertainty metrics and predictive performance were optimal when leveraging the full spectrum of preoperative and intraoperative physiologic time-series data as predictor variables in an efficient multi-task deep learning model. Uncertainty-aware deep learning may have utility for understanding the probability that a prediction deviates substantially from usual predictions and represents a rare, major prediction error. Integrated gradients interpretability mechanisms identified biologically plausible important features. The accurate, interpretable, uncertainty-aware predictions presented herein require further investigation regarding their potential to augment surgical decision-making during preoperative and immediate postoperative phases of care.

## Methods

All analyses were performed on a retrospective, single-center, longitudinal cohort of surgical patients that included data from both preoperative and intraoperative phases of care. We used deep learning, random forest, and XGBoost models to predict the onset of nine major postoperative complications following surgery with three primary objectives: (1) compare deep learning techniques with random forest and XGBoost models in predicting postoperative complications, (2) compare deep learning predictions made at two phases of perioperative care: immediately before surgery (using preoperative data alone, referred to henceforth as preoperative prediction), and immediately after surgery by two different methods: (a) using intraoperative data alone (referred to henceforth as intraoperative prediction), and (b) using both preoperative and intraoperative data (referred to henceforth as postoperative prediction), and (3) explore the potential benefits of three novel deep learning techniques: (a) multi-task learning by training a single deep learning model on several postoperative complications compared with training separate models for each individual complication, (b) model interpretability with integrated gradients, and (c) model uncertainty-awareness by calculating variance across predictions.

The University of Florida Institutional Review Board and Privacy Office approved this study as an exempt study with waiver of informed consent (IRB # 201600223). Recommendations were followed from both Transparent Reporting of a multivariable prediction model for Individual Prognosis Or Diagnosis (TRIPOD^39^) guidelines and from best practices for prediction modeling from Leisman et al.^40^ All methods were performed in accordance with relevant guidelines and regulations.

### Data source

The University of Florida Integrated Data Repository was used as an honest broker to build a longitudinal dataset representing patients admitted to University of Florida Health between June 1st, 2014, and September 20th, 2020, who were at least 18 years of age and underwent at least one surgical procedure during hospitalization. The dataset was constructed by integrating electronic health records with other clinical, administrative, and public databases^9^. The resulting dataset included information on patient demographics, laboratory values, vital signs, diagnoses, medications, blood product administration, procedures, and clinical outcomes, as well as detailed intraoperative physiologic and monitoring data.

### Predictors

Our final cohort included electronic health record data from both before and during surgery. Preoperative models were trained on data available between 1 year prior to surgery and the day of surgery, prior to surgery start time (i.e., preoperative features alone). Intraoperative models were trained on data created during the surgical procedure (i.e., intraoperative features alone). Postoperative models were trained on data available between 1 year prior to surgery through the end of the surgical procedure (i.e., both preoperative and intraoperative features).

We identified 402 preoperative features, including demographic and socioeconomic indicators, planned procedure and provider information, Charlson comorbidities, and summary statistics of select medications, laboratory tests, and physiological measurements (e.g., vital signs such as heart rate and blood pressure) taken prior to a surgical procedure over 1-year and 1-week time windows. We calculated Charlson comorbidity indices using International Classification of Diseases (ICD) codes^41^. We modeled procedure types on ICD-9-CM codes with a forest structure in which nodes represent groups of procedures, roots represent the most general groups of procedures, and leaf nodes represent specific procedures. Medications were derived from RxNorm codes grouped into drug classes as previously described.

Intraoperative data consisted of 14 physiological measurements taken during surgery: systolic blood pressure, diastolic blood pressure, mean arterial pressure, heart rate, blood oxygen saturation (SpO2), fraction of inspired oxygen (FiO2), end-tidal carbon dioxide (EtCO2), tidal volume, respiration rate, peak inspiratory pressure (PIP), minimum alveolar concentration (MAC), temperature, urine output, and operative blood loss. These variables were presented to deep learning models as variable-length multivariate time series. For random forest and XGBoost models, a set of 49 statistical features were extracted from each encounter’s intraoperative measurements. Supplementary Table S6 summarizes all input features and relevant preprocessing procedures.

### Participants

We excluded patients with intraoperative mortality or who were missing the variables necessary to classify postoperative complications. If a single patient’s hospital encounter included more than one surgery, only the first surgery during that encounter was included in our analyses. Our final dataset included 56,242 patients who underwent 67,481 surgeries. Supplementary Fig. S5 illustrates derivation of the study population and cohort selection criteria.

### Outcomes

We used several different machine learning methods to model the risk of nine postoperative complications: prolonged intensive care unit stay (greater than 48 h), prolonged mechanical ventilation requirement (greater than 48 h), neurological complications, cardiovascular complications, acute kidney injury, sepsis, venous thromboembolism, wound complications, and in-hospital mortality.

### Sample size

We chronologically divided our perioperative cohort into a development set of 47,188 surgeries occurring between June 1st, 2014, through November 26th, 2018, and a validation set of 20,293 surgeries occurring between November 27th, 2018, through September 20th, 2020. All models were trained using the development patient cohort; all results were reported for the validation patient cohort (Supplementary Fig. S5). While training deep learning models, we used 10% of encounters from the development cohort for early stopping.

Using a validation cohort of 20,293 surgeries, the overall sample size allows for a maximum width of the 95% confidence interval for area under the receiver operating characteristic curve (AUROC) to be between 0.01 and 0.03 for postoperative complications with prevalence ranging between 5.4 and 33.3% for AUROC of 0.80 or higher. The sample size allows for a maximum width of 0.06 for hospital mortality given 1.6% prevalence.

### Predictive analytic workflow

The postoperative models update preoperative risk predictions using data collected during surgery. This workflow emulates clinical scenarios in which patients’ preoperative information is enriched by the influx of new data from the operating room. The model consists of two main preoperative and intraoperative layers, each containing a data transformer core and a data analytics core^9^. The data transformer integrates data from multiple sources, including EHR data with ZIP code links to US Census data for patient neighborhood characteristics and distance from the hospital. The data transformer then performs preprocessing and feature transformation steps to optimize the data for analysis.

The 402 preoperative features contained 341 continuous features, 42 binary features, and 19 nominal features. Of the 19 nominal features, 13 contained fewer than 5 levels and were one-hot encoded as zero vectors of dimension equal to number of levels, with level indicators equal to one. The remaining six nominal features (ZIP code, attending surgeon, primary procedure, scheduled operating room, surgery type, and surgeon specialty) were represented as unique integer identifiers ranging from zero to the number of levels minus one. Implicit variable representations were learned as part of the model training process. Continuous preoperative feature observations that fell below the 1st or above the 99th percentiles were capped to the 1st and 99th percentile values, respectively. Temporal preoperative features denoting the day and month of admission were transformed into two individual continuous features each through the use of sinusoidal functions based on the respective frequency of days or months, which encoded relative differences between time points (e.g., Sunday is close to Monday, and December is close to January).

Intraoperative measurements were identified as those falling between anesthesia start and stop times for a given procedure. Fixed-interval multivariate physiological time series were constructed for each procedure by resampling measured values to a frequency of one minute, which represented the highest recorded frequency across all intraoperative features. For a given surgical procedure which had at least one measurement of a given feature, any gaps in that feature’s time series were imputed via linear interpolation in both directions. As surgeries vary in duration, each sample included a multivariate time series of length *T* minutes. Blood loss sum, urine output sum, and duration of surgery were included as static postoperative features.

Missing continuous features were imputed with the median of each feature value in the development cohort. For static preoperative descriptors, this represented a single number; for intraoperative time series, this was only performed when a single feature value did not exist, and the median value was imputed at every one-minute time step for the full duration of surgery. Missing preoperative nominal features were replaced with a distinct “missing” category.

To preserve patterns of missingness which may be informative^42^, for each sample we derived a preoperative binary presence mask over all continuous and binary input variables that indicated whether a given value was observed or imputed. These missingness indicators were concatenated with their respective original measurements. For a given cohort set of size *N* encounters, initial continuous and binary preoperative features were represented as a matrix of descriptors *P*^*N*×383^. With a missingness mask of size $P_{mask}^{N \times 383}$, concatenation resulted in a final continuous and binary preoperative feature set of 766 numerical preoperative descriptors for each sample. Nominal preoperative features did not require a missingness mask, as missing values were transformed into a distinct categorical level. The 13 nominal variables that were one-hot encoded were concatenated with the above numerical preoperative representation, and the 6 nominal features with greater than 5 levels were internally embedded by the model. Multivariate time series missingness masks were computed and concatenated at each one-minute intraoperative timestep; for a single surgical time series *x*^*T*×12^ of length *T* including our 12 temporal physiological measurements, the concatenation of these per-timestep masks resulted in a final input time series *x*^*T*×24^ of 24 intraoperative predictors at each timestep. All continuous input variables, both preoperative and intraoperative, were z-normalized to zero mean and unit variance based on values from the development set.

Following these processing steps, each surgical encounter was represented by four distinct sets of variables: a set of numerical preoperative features, a set of nominal preoperative features to be internally embedded by the model, a multivariate time series of length *T* composed of physiological measurements, and a set of static surgical features collected at the end of surgery. The length of intraoperative time series varied depending on surgery duration, and our deep learning models were designed to process the full scope of intraoperative physiological measurements.

In the data analytics core, deep learning, random forest, and XGBoost models were trained to predict nine postoperative complications following a surgical procedure. Clinically, predictions made by preoperative models can inform patients, caregivers, and surgeons regarding risks of undergoing surgery, and estimate the utility of risk reduction strategies for specific complications (e.g., preoperative smoking cessation, perioperative renal protection bundles, and wound closure techniques). Intraoperative events can influence risk for complications (e.g., operative blood loss requiring allogenic blood transfusion increases risk for septic complications and intraoperative hypotension increases risk for acute kidney injury). Therefore, we generated intraoperative models to predict complications using data obtained during surgery. At the end of surgery, clinicians must reassess the patient’s prognosis, convey this information to the patient and their caregivers, and make clinical management decisions accordingly (e.g., a patient at high risk for cardiovascular complications may benefit from postoperative admission to an intensive care unit or continuous cardiac telemetry on a general hospital ward). At the end of surgery, it seems prudent to consider both baseline preoperative risk as well as the potential influence of intraoperative events to make updated predictions of postoperative complications. This is accomplished by our postoperative models.

**Figure 3:**
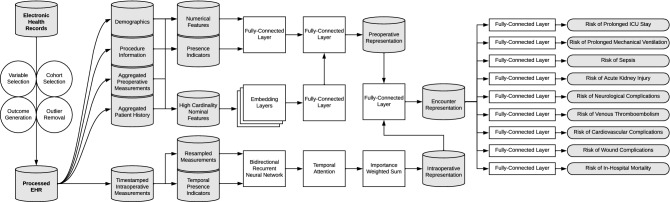
Data processing pipeline and deep learning model architecture. Patient-level input variables were split into static preoperative data and temporal intraoperative data. Preoperative variables were split into continuous, binary, and high-cardinality features and followed variable-specific preprocessing procedures. Deep learning model architecture utilized a data fusion design combining latent representations of high-frequency intraoperative data (from a bidirectional recurrent neural network) and static preoperative patient data (from fully connected layers) for eventual multi-task prediction of nine postoperative complications.

As a technical explanation of deep learning fundamentals is beyond the scope of this study, we refer interested readers to the comprehensive work by Goodfellow et al.^43^ Our final postoperative deep learning model can be conceptualized as a composition of two sub-models: one for processing preoperative features, and one for processing intraoperative features. Reported preoperative results (i.e., predicting postoperative complications using preoperative features alone) were obtained by only using the data representation from the preoperative sub-model; likewise, reported intraoperative results were obtained by only using the data representation from the intraoperative sub-model. The postoperative model used a transformed concatenation of both preoperative and intraoperative data representations (Fig. 3).

The preoperative sub-model was composed of a dual pipeline for processing and representing numerical features and nominal features with greater than 5 levels. A representation of all six index-encoded nominal input features was obtained by concatenating individual nominal feature representations, each of which were the result of a learned, multidimensional per-feature embedding lookup table, and passing the concatenated result through a fully connected layer. A representation of all numerical preoperative variables was obtained by passing the input features through a fully connected layer. A complete preoperative encounter representation was obtained by concatenating both continuous and nominal input feature representations and passing the result through a final fully connected layer.

In the multi-task setting, this preoperative data representation was passed through nine branches corresponding to our nine postoperative complication outcomes. Each branch contained one outcome-specific fully connected layer followed by a sigmoid activation function to produce a per-outcome prediction score, interpreted as the probability of a preoperative patient developing a given postoperative complication.

The primary driving force behind the intraoperative sub-model was a bidirectional recurrent neural network (RNN) with gated recurrent units (GRU). A patient’s intraoperative time series was passed through the RNN twice, once in chronological order and once in reverse order. Time step representations were generated by concatenating the RNN hidden states from the forward and backward passes. An attention mechanism was applied to the bidirectional sequence representations. Briefly, an attention mechanism for classification allows a model to assign importance scores to individual timesteps of a representation sequence such that the importance-weighted sequence is summed into a single context vector that is an optimal representation for a given predictive task. Attention allows a model to learn to focus exclusively on timesteps that are important for classification decisions. In our multi-task model, we implemented a separate attention mechanism for each of the nine postoperative complications. Using a shared representation of an intraoperative sequence from the RNN, each attention component formulated a separate perspective of the sequence aligned with each outcome of interest.

Our complete deep learning model, which we refer to as the postoperative model, includes both the preoperative and intraoperative sub-models described in this section. The postoperative model is trained end-to-end and consists of concatenating both the static preoperative representation (the output of the preoperative sub-model) with the outcome-specific intraoperative representation (the output of the intraoperative sub-model for a given outcome) and passing this combined feature representation through the same set of nine classification branches as the sub-models.

In our experiments and reported results, we use a nominal preoperative variable embedding size of 64, fully connected layers size of 64 (except for final task output layers, which have size 1), hidden dimension of 64, Adam optimizer with learning rate of 0.001, L2 regularization of 0.01, batch size of 64, RELU activation, and patience of 4 used for early stopping based on the validation data set. Given the large number of models and experimental settings contained in this work (in addition to hardware limitations), we do not perform an exhaustive hyperparameter search; instead, we selected these values based on our previously successful implementations of recurrent neural networks for clinical patient data in prior work^18,44^.

To determine whether the deep learning models offered a performance advantage over traditional predictive analytic methods, we assessed the performance of baseline random forest and XGBoost classifiers using the same preoperative and intraoperative input feature sets as the deep learning models, with predictions made at the same time points. Nominal preoperative features, which were index-encoded before passing through the deep model, were instead one-hot encoded before feeding into the baseline models. Intraoperative time series were fed to the baseline models by way of 49 summary statistics, capturing static attributes and patterns of variability for each variable. These features are described in Supplementary Table S6.

To account for class imbalance among the nine postoperative complication outcomes, both deep learning and baseline models were trained using outcome-specific class weights that were inversely proportional to their respective frequencies in the training set. Functionally, this ensures greater model focus on minority class samples.

The postoperative complication predictions from all deep neural networks trained under each surgical phase (preoperative, intraoperative, postoperative) and training scheme (individual models, multi-task learning) were analyzed with Monte Carlo dropout, approximating Bayesian inference and providing a quantitative measure of uncertainty for neural network predictions^45^. By enabling randomized dropout during model inference and aggregating resulting predictions over several experimental trials, a pseudo model ensemble is generated with partially randomized neural network connections. In our experiments, we perform 100 trials with stochastic dropout applied during inference and compute the mean complication risk and resulting prediction variance as a measure of model uncertainty.

We apply the method of integrated gradients to our final postoperative multi-task model to illuminate specific input features that yielded the largest impacts on predicting each of the nine complication outcomes. A complete discussion of this technique is beyond the scope of this study; we refer interested readers to the work of Sundarajan et al.^46^. Briefly, integrated gradients is a comparative technique for local interpretability, centered around the analysis of model outputs based on a given input and corresponding baseline values, and assigns attributions values to every input feature. In theory, features most influential to a given prediction will receive larger attribution values, and taken over an entire population, this can reveal the importance of certain features which drive the model predictions. We use a zero-vector reference value for such computations, and as all variables are Z-normalized to zero mean and unit variance; such a reference can be viewed as the per-variable mean value across the entire cohort.

### Model validation

All models were trained on the development set of 47,188 surgeries occurring between June 1st, 2014, through November 26th, 2018. Models were evaluated on the validation set of 20,293 surgical procedures occurring between November 27th, 2018, through September 20th, 2020. For each model performance metric, ninety-five percent nonparametric confidence intervals were calculated using 1000 bootstrapped samples with replacement.

### Model performance

Model performance was evaluated by sensitivity, specificity, positive predictive value (PPV), negative predictive value (NPV), accuracy, area under the precision-recall curve (AUPRC), and area under the receiver operating characteristic curve (AUROC). Reported metrics include class predictions based on Youden’s index threshold on predicted risk scores, which maximizes sensitivity and specificity, as the cutoff point for low versus high risk^47^.

When predicting rare events, models can exhibit deceivingly high accuracy by predicting negative outcomes in predominantly negative datasets^48^. False negative predictions of postoperative complications may be especially detrimental because patients, caregivers, and surgeons could unknowingly agree to perform prohibitively high-risk surgery, miss opportunities to mitigate preventable harm through prehabilitation and other risk-reduction strategies, and under-triage high-risk patients to general hospital wards with infrequent monitoring, when close monitoring in an intensive care unit would be safer. Therefore, model performance was evaluated by calculating area under the precision-recall curve (AUPRC), which is adept at evaluating the performance of models predicting rare events^49^. In addition, Net Reclassification Improvement (NRI) indices were used to describe and quantify correct and incorrect reclassifications by deep learning models^50^. For all performance metrics, we used bootstrap sampling and non-parametric methods to obtain 95% confidence intervals.

## Supplementary Information

Supplementary Information.

## Data Availability

Data is available from the University of Florida Institutional Data Access/Ethics Committee for researchers who meet the criteria for access to confidential data and may require additional IRB approval.
