# Supplementary material for: Dynamic predictions of postoperative complications from explainable, uncertainty-aware, and multi-task deep neural networks
Source: Sci Rep. 2023 Jan 21;13:1224. doi: 10.1038/s41598-023-27418-5 (PMC9867692; doi:10.1038/s41598-023-27418-5)
Supplement: Supplementary file 1 — Supplementary Information. [file 41598_2023_27418_MOESM1_ESM.docx]

**Dynamic Predictions of Postoperative Complications from Explainable, Uncertainty-Aware, and Multi-Task Deep Neural Networks**

Benjamin Shickel, PhD ^1,6^, Tyler J. Loftus, MD ^2,6^, Matthew Ruppert, BS ^1,3,6^, Gilbert R. Upchurch Jr., MD ^2^, Tezcan Ozrazgat-Baslanti, PhD ^1,3,6^, Parisa Rashidi, PhD ^1,4,5,6^, Azra Bihorac, MD, MS ^1,3,6,*^

^1^ Department of Medicine, University of Florida, Gainesville, FL, 32611, USA

^2^ Department of Surgery, University of Florida, Gainesville, FL, 32611, USA

^3^ Precision and Intelligent Systems in Medicine (PRISMAp), University of Florida, Gainesville, FL, 32611, USA

^4^ Department of Biomedical Engineering, University of Florida, Gainesville, FL, 32611, USA

^5^ Intelligent Health Lab (i-Heal), University of Florida, Gainesville, FL, 32611, USA

^6^ Intelligent Critical Care Center (IC^3^), University of Florida, Gainesville, FL, 32611, USA

**SUPPLEMENTARY TABLES**

**Supplementary Table S1. Summary of admission variables and prevalence of postoperative complications.**

|  | **Development Cohort** (6/1/2014 - 11/26/2018) | **Validation Cohort** (11/27/2018 - 9/20/2020) |
| --- | --- | --- |
| **Patients, n** | 38621 | 17621 |
| **Hospital encounters, n** | 47188 | 20293 |
| **Length of stay, days, median (25th, 75th)** | 4.1 (2.2, 7.9) | 4.3 (2.2, 8.4) |
| **Length of surgery, hours, median (25th, 75th)** | 3.1 (2.2, 4.6) | 3.2 (2.3, 4.7) |
| **Surgery lead time, hours, median (25th, 75th)** | 2.7 (2.0, 10.1) | 2.9 (2.1, 11.9) |
| **Admission Source, n (%)** |  |  |
| Non-Transfer | 39986 (84.7%) | 16976 (83.7%) |
| Transfer | 7202 (15.3%) | 3317 (16.3%) |
| **Emergent Admission, n (%)** |  |  |
| Non-Emergent | 30482 (64.6%) | 12802 (63.1%) |
| Emergent | 16706 (35.4%) | 7491 (36.9%) |
| **Admission Day, n (%)** |  |  |
| Weekday | 36811 (78.0%) | 15690 (77.3%) |
| Weekend | 10377 (22.0%) | 4603 (22.7%) |
| **Admission Time, n (%)** |  |  |
| Daytime | 25100 (53.2%) | 10587 (52.2%) |
| Nighttime | 22088 (46.8%) | 9706 (47.8%) |
| **Anesthesia Type, n (%)** |  |  |
| General | 42963 (91.0%) | 18741 (92.4%) |
| Local | 4225 (9.0%) | 1552 (7.6%) |
| **Admission Type, n (%)** |  |  |
| Medicine | 20893 (44.3%) | 8277 (40.8%) |
| Surgery | 17899 (37.9%) | 7806 (38.5%) |
| Other | 8396 (17.8%) | 4210 (20.7%) |
| **Surgery Type, n (%)** |  |  |
| Orthopaedic | 11831 (25.1%) | 5092 (25.1%) |
| Neurosurgery | 6757 (14.3%) | 3312 (16.3%) |
| Urologic | 4272 (9.1%) | 1459 (7.2%) |
| Vascular | 3731 (7.9%) | 1809 (8.9%) |
| Otolaryngology | 3261 (6.9%) | 1178 (5.8%) |
| Cardiothoracic | 3363 (7.1%) | 1038 (5.1%) |
| Gastrointestinal | 2935 (6.2%) | 1199 (5.9%) |
| Gynecologic | 2051 (4.3%) | 631 (3.1%) |
| Oncology | 1827 (3.9%) | 496 (2.4%) |
| Plastic | 1118 (2.4%) | 420 (2.1%) |
| Burn | 939 (2.0%) | 354 (1.7%) |
| Transplantation | 657 (1.4%) | 225 (1.1%) |
| Other | 4446 (9.4%) | 3080 (15.2%) |
| **Scheduled Postoperative Location, n (%)** |  |  |
| Non-ICU | 37143 (78.7%) | 18020 (88.8%) |
| ICU | 10045 (21.3%) | 2273 (11.2%) |
| **Postoperative Complications, n (%)** |  |  |
| Prolonged ICU Stay (> 2 Days) | 12980 (27.5%) | 6765 (33.3%) |
| Prolonged Mechanical Ventilation (> 2 Days) | 3512 (7.4%) | 1574 (7.8%) |
| Wound Complications | 6782 (14.4%) | 4347 (21.4%) |
| Neurological Complications | 7273 (15.4%) | 4107 (20.2%) |
| Cardiovascular Complications | 5655 (12.0%) | 3301 (16.3%) |
| Sepsis | 3445 (7.3%) | 1775 (8.7%) |
| Acute Kidney Injury | 6894 (14.6%) | 3438 (16.9%) |
| Venous Thromboembolism | 2008 (4.3%) | 1101 (5.4%) |
| In-Hospital Mortality | 788 (1.7%) | 321 (1.6%) |

**Supplementary Table S2. Summary of sociodemographic variables.**

|  | **Development Cohort** (6/1/2014 - 11/26/2018) | **Validation Cohort** (11/27/2018 - 9/20/2020) |
| --- | --- | --- |
| **Patients, n** | 38621 | 17621 |
| **Hospital encounters, n** | 47188 | 20293 |
| **Age, years, median (25th, 75th)** | 59.0 (45.0, 69.0) | 61.0 (47.0, 71.0) |
| **Body mass index, median (25th, 75th)** | 28.1 (24.0, 33.3) | 28.1 (24.0, 33.3) |
| **Sex, n (%)** |  |  |
| Female | 23716 (50.3%) | 10005 (49.3%) |
| Male | 23472 (49.7%) | 10288 (50.7%) |
| **Ethnicity, n (%)** |  |  |
| Non-Hispanic | 44255 (93.8%) | 18817 (92.7%) |
| Hispanic | 2142 (4.5%) | 987 (4.9%) |
| Unknown | 791 (1.7%) | 489 (2.4%) |
| **Race, n (%)** |  |  |
| White | 37047 (78.5%) | 15942 (78.6%) |
| African American | 6562 (13.9%) | 2759 (13.6%) |
| Other/Unknown | 3579 (7.6%) | 1592 (7.8%) |
| **Language, n (%)** |  |  |
| English | 46342 (98.2%) | 19926 (98.2%) |
| Other | 846 (1.8%) | 367 (1.8%) |
| **Marital Status, n (%)** |  |  |
| Married | 22574 (47.8%) | 9586 (47.2%) |
| Single | 17040 (36.1%) | 7665 (37.8%) |
| Divorced | 7218 (15.3%) | 2902 (14.3%) |
| Unknown | 356 (0.8%) | 140 (0.7%) |
| **Smoking Status, n (%)** |  |  |
| Never | 20244 (42.9%) | 8986 (44.3%) |
| Former | 15948 (33.8%) | 6923 (34.1%) |
| Current | 8711 (18.5%) | 3485 (17.2%) |
| Unknown | 2285 (4.8%) | 899 (4.4%) |
| **Insurance, n (%)** |  |  |
| Medicare | 20859 (44.2%) | 9701 (47.8%) |
| Private | 14677 (31.1%) | 5740 (28.3%) |
| Medicaid | 7987 (16.9%) | 3023 (14.9%) |
| Uninsured | 3665 (7.8%) | 1829 (9.0%) |
| **Admission comorbidities, n (%)** |  |  |
| Myocardial infarction | 3253 (6.9%) | 1525 (7.5%) |
| Congestive heart failure | 6923 (14.7%) | 3505 (17.3%) |
| Peripheral vascular disease | 9569 (20.3%) | 4803 (23.7%) |
| Cerebrovascular disease | 7691 (16.3%) | 3537 (17.4%) |
| Chronic pulmonary disease | 14579 (30.9%) | 6715 (33.1%) |
| Metastatic carcinoma | 4474 (9.5%) | 1961 (9.7%) |
| Cancer | 13330 (28.2%) | 5366 (26.4%) |
| Liver disease | 6670 (14.1%) | 3132 (15.4%) |
| Diabetes | 10803 (22.9%) | 4806 (23.7%) |
| Hypertension | 29015 (61.5%) | 13346 (65.8%) |
| Hypothyroidism | 8196 (17.4%) | 3714 (18.3%) |
| Valvular disease | 5987 (12.7%) | 3316 (16.3%) |
| Coagulopathy | 6117 (13.0%) | 2709 (13.3%) |
| Obesity | 14855 (31.5%) | 8696 (42.9%) |
| Weight loss | 6445 (13.7%) | 3154 (15.5%) |
| Fluid/electrolyte disorders | 13266 (28.1%) | 7605 (37.5%) |
| Chronic anemia | 8762 (18.6%) | 5086 (25.1%) |
| Alcohol or drug abuse | 7102 (15.1%) | 3189 (15.7%) |
| Depression | 13001 (27.6%) | 6021 (29.7%) |
| **Unique diagnosis codes, n, median (25th, 75th)** | 40.0 (20.0, 88.0) | 47.0 (24.0, 108.0) |
| **Charlson comorbidity index, median (25th, 75th)** | 4.0 (2.0, 6.0) | 4.0 (2.0, 6.0) |
| **Neighborhood Characteristics, median (25th, 75th)** |  |  |
| Total population, n | 17583.0 (10722.5, 27063.0) | 17583.0 (10725.0, 27063.0) |
| Distance to hospital, km | 42.9 (22.1, 80.7) | 43.6 (22.3, 78.5) |
| Median income, dollars | 40528.0 (35244.0, 48493.0) | 40532.0 (35714.5, 48457.8) |
| Poverty rate | 19.1 (13.5, 25.0) | 18.8 (13.4, 24.9) |
| African American population proportion | 0.1 (0.0, 0.2) | 0.1 (0.0, 0.2) |
| Hispanic population proportion | 0.1 (0.0, 0.1) | 0.1 (0.0, 0.1) |
| **Rural/Urban, n (%)** |  |  |
| Urban | 30724 (65.1%) | 13119 (64.6%) |
| Rural | 16392 (34.7%) | 7132 (35.1%) |
| Unknown | 72 (0.2%) | 42 (0.2%) |

**Supplementary Table S3. Summary of medication and laboratory history variables.**

|  | **Development Cohort** (6/1/2014 - 11/26/2018) | **Validation**  **Cohort** (11/27/2018 - 9/20/2020) |
| --- | --- | --- |
| **Patients, n** | 38621 | 17621 |
| **Hospital encounters, n** | 47188 | 20293 |
| **Received medications in past year, n (%)** |  |  |
| ACE Inhibitors | 4253 (9.0%) | 1677 (8.3%) |
| Aminoglycosides | 1631 (3.5%) | 801 (3.9%) |
| Antiemetics | 13282 (28.1%) | 5848 (28.8%) |
| Aspirin | 5902 (12.5%) | 2576 (12.7%) |
| Beta Blockers | 7164 (15.2%) | 3272 (16.1%) |
| Bicarbonates | 4672 (9.9%) | 2513 (12.4%) |
| Corticosteroids | 6692 (14.2%) | 3676 (18.1%) |
| Diuretics | 4731 (10.0%) | 1931 (9.5%) |
| NSAIDS | 6531 (13.8%) | 3239 (16.0%) |
| Vasopressors/Inotropes | 9187 (19.5%) | 4267 (21.0%) |
| Statins | 3803 (8.1%) | 1847 (9.1%) |
| Vancomycin | 4803 (10.2%) | 2445 (12.0%) |
| Nephrotoxic | 5338 (11.3%) | 2184 (10.8%) |
| Total medications, n, median (25th, 75th) | 0.0 (0.0, 3.0) | 0.0 (0.0, 3.0) |
| **Urea nitrogen/creatinine, ratio** |  |  |
| Minimum (0-7 days prior), median (25th, 75th) | 15.6 (11.9, 20.0) | 16.4 (12.5, 21.0) |
| Maximum (0-7 days prior), median (25th, 75th) | 17.6 (13.8, 22.8) | 18.6 (14.4, 23.8) |
| Average (0-7 days prior), median (25th, 75th) | 16.7 (13.0, 21.3) | 17.5 (13.7, 22.2) |
| Variance (0-7 days prior), median (25th, 75th) | 0.0 (0.0, 2.5) | 0.0 (0.0, 2.6) |
| Measurements (0-7 days prior), n, median (25th, 75th) | 4.0 (0.0, 8.0) | 5.0 (0.0, 10.0) |
| Minimum (8-365 days prior), median (25th, 75th) | 13.7 (10.2, 17.9) | 14.3 (10.8, 18.6) |
| Maximum (8-365 days prior), median (25th, 75th) | 19.8 (15.5, 25.6) | 21.0 (16.3, 27.3) |
| Average (8-365 days prior), median (25th, 75th) | 16.8 (13.4, 20.9) | 17.7 (14.2, 22.2) |
| Variance (8-365 days prior), median (25th, 75th) | 2.9 (0.0, 13.2) | 3.5 (0.0, 14.7) |
| Measurements (8-365 days prior), n, median (25th, 75th) | 4.0 (0.0, 15.0) | 5.0 (0.0, 19.0) |
| **Hemoglobin, g/dL** |  |  |
| Minimum (0-7 days prior), median (25th, 75th) | 12.6 (10.8, 14.0) | 12.5 (10.8, 13.9) |
| Maximum (0-7 days prior), median (25th, 75th) | 13.2 (11.8, 14.5) | 13.1 (11.7, 14.4) |
| Average (0-7 days prior), median (25th, 75th) | 12.9 (11.3, 14.2) | 12.8 (11.2, 14.1) |
| Variance (0-7 days prior), median (25th, 75th) | 0.4 (0.2, 1.1) | 0.4 (0.1, 1.0) |
| Measurements (0-7 days prior), n, median (25th, 75th) | 1.0 (0.0, 2.0) | 1.0 (0.0, 2.0) |
| Minimum (8-365 days prior), median (25th, 75th) | 12.1 (9.9, 13.7) | 12.1 (9.8, 13.6) |
| Maximum (8-365 days prior), median (25th, 75th) | 13.7 (12.6, 14.8) | 13.7 (12.5, 14.7) |
| Average (8-365 days prior), median (25th, 75th) | 12.7 (11.1, 14.0) | 12.7 (11.1, 13.9) |
| Variance (8-365 days prior), median (25th, 75th) | 0.9 (0.3, 1.9) | 0.8 (0.3, 1.7) |
| Measurements (8-365 days prior), n, median (25th, 75th) | 1.0 (0.0, 4.0) | 1.0 (0.0, 4.0) |
| **Leukocytes, thou/uL** |  |  |
| Minimum (0-7 days prior), median (25th, 75th) | 7.6 (6.0, 10.0) | 7.5 (5.8, 9.9) |
| Maximum (0-7 days prior), median (25th, 75th) | 8.7 (6.7, 12.1) | 8.6 (6.5, 11.9) |
| Average (0-7 days prior), median (25th, 75th) | 8.2 (6.4, 10.9) | 8.1 (6.3, 10.8) |
| Variance (0-7 days prior), median (25th, 75th) | 1.8 (0.5, 6.2) | 1.6 (0.4, 5.1) |
| Measurements (0-7 days prior), n, median (25th, 75th) | 1.0 (0.0, 2.0) | 1.0 (0.0, 2.0) |
| Minimum (8-365 days prior), median (25th, 75th) | 6.4 (5.0, 8.0) | 6.1 (4.8, 7.7) |
| Maximum (8-365 days prior), median (25th, 75th) | 9.0 (6.9, 12.6) | 8.9 (6.7, 12.7) |
| Average (8-365 days prior), median (25th, 75th) | 7.7 (6.2, 9.7) | 7.5 (6.0, 9.5) |
| Variance (8-365 days prior), median (25th, 75th) | 2.8 (0.8, 7.7) | 2.8 (0.8, 7.6) |
| Measurements (8-365 days prior), n, median (25th, 75th) | 1.0 (0.0, 3.0) | 1.0 (0.0, 3.0) |
| **Erythrocytes, million/uL** |  |  |
| Minimum (0-7 days prior), median (25th, 75th) | 4.2 (3.7, 4.7) | 4.2 (3.7, 4.6) |
| Maximum (0-7 days prior), median (25th, 75th) | 4.4 (4.0, 4.8) | 4.3 (3.9, 4.8) |
| Average (0-7 days prior), median (25th, 75th) | 4.3 (3.8, 4.7) | 4.3 (3.8, 4.7) |
| Variance (0-7 days prior), median (25th, 75th) | 0.0 (0.0, 0.1) | 0.0 (0.0, 0.1) |
| Measurements (0-7 days prior), n, median (25th, 75th) | 1.0 (0.0, 2.0) | 1.0 (0.0, 2.0) |
| Minimum (8-365 days prior), median (25th, 75th) | 4.1 (3.4, 4.6) | 4.1 (3.4, 4.5) |
| Maximum (8-365 days prior), median (25th, 75th) | 4.6 (4.2, 4.9) | 4.5 (4.2, 4.9) |
| Average (8-365 days prior), median (25th, 75th) | 4.3 (3.8, 4.7) | 4.3 (3.8, 4.7) |
| Variance (8-365 days prior), median (25th, 75th) | 0.1 (0.0, 0.2) | 0.1 (0.0, 0.2) |
| Measurements (8-365 days prior), n, median (25th, 75th) | 1.0 (0.0, 3.0) | 1.0 (0.0, 3.0) |
| **Hematocrit, %** |  |  |
| Minimum (0-7 days prior), median (25th, 75th) | 38.1 (33.1, 41.8) | 37.4 (32.5, 41.2) |
| Maximum (0-7 days prior), median (25th, 75th) | 39.8 (35.7, 43.1) | 39.2 (35.0, 42.7) |
| Average (0-7 days prior), median (25th, 75th) | 38.9 (34.5, 42.3) | 38.3 (33.8, 41.8) |
| Variance (0-7 days prior), median (25th, 75th) | 0.0 (0.0, 2.5) | 0.0 (0.0, 2.5) |
| Measurements (0-7 days prior), n, median (25th, 75th) | 2.0 (0.0, 4.0) | 2.0 (0.0, 4.0) |
| Minimum (8-365 days prior), median (25th, 75th) | 36.7 (30.3, 41.1) | 36.1 (29.6, 40.4) |
| Maximum (8-365 days prior), median (25th, 75th) | 41.3 (38.2, 44.4) | 40.7 (37.6, 43.6) |
| Average (8-365 days prior), median (25th, 75th) | 38.5 (34.2, 42.0) | 38.0 (33.4, 41.3) |
| Variance (8-365 days prior), median (25th, 75th) | 2.8 (0.0, 10.9) | 2.6 (0.0, 10.3) |
| Measurements (8-365 days prior), n, median (25th, 75th) | 2.0 (0.0, 6.0) | 2.0 (0.0, 6.0) |
| **Erythrocyte mean corpuscular volume, fL** |  |  |
| Minimum (0-7 days prior), median (25th, 75th) | 89.8 (85.9, 93.6) | 89.1 (85.1, 92.9) |
| Maximum (0-7 days prior), median (25th, 75th) | 90.6 (86.6, 94.4) | 89.8 (85.8, 93.6) |
| Average (0-7 days prior), median (25th, 75th) | 90.2 (86.3, 94.0) | 89.4 (85.4, 93.2) |
| Variance (0-7 days prior), median (25th, 75th) | 0.6 (0.2, 1.6) | 0.4 (0.1, 1.0) |
| Measurements (0-7 days prior), n, median (25th, 75th) | 1.0 (0.0, 2.0) | 1.0 (0.0, 2.0) |
| Minimum (8-365 days prior), median (25th, 75th) | 88.8 (84.7, 92.7) | 88.1 (83.7, 92.0) |
| Maximum (8-365 days prior), median (25th, 75th) | 91.6 (87.7, 95.7) | 90.7 (86.9, 94.7) |
| Average (8-365 days prior), median (25th, 75th) | 90.2 (86.4, 94.0) | 89.4 (85.4, 93.1) |
| Variance (8-365 days prior), median (25th, 75th) | 2.1 (0.7, 5.1) | 1.7 (0.6, 4.6) |
| Measurements (8-365 days prior), n, median (25th, 75th) | 1.0 (0.0, 3.0) | 1.0 (0.0, 3.0) |
| **Erythrocyte mean corpuscular hemoglobin concentration, g/dL** |  |  |
| Minimum (0-7 days prior), median (25th, 75th) | 32.9 (31.9, 33.8) | 33.3 (32.7, 34.0) |
| Maximum (0-7 days prior), median (25th, 75th) | 33.6 (32.8, 34.4) | 33.7 (33.0, 34.3) |
| Average (0-7 days prior), median (25th, 75th) | 33.3 (32.4, 34.0) | 33.5 (32.9, 34.1) |
| Variance (0-7 days prior), median (25th, 75th) | 0.2 (0.1, 0.4) | 0.2 (0.1, 0.3) |
| Measurements (0-7 days prior), n, median (25th, 75th) | 2.0 (0.0, 2.0) | 1.0 (0.0, 2.0) |
| Minimum (8-365 days prior), median (25th, 75th) | 32.4 (31.3, 33.4) | 33.0 (32.2, 33.7) |
| Maximum (8-365 days prior), median (25th, 75th) | 33.8 (33.0, 34.5) | 34.1 (33.3, 34.8) |
| Average (8-365 days prior), median (25th, 75th) | 33.1 (32.2, 33.8) | 33.5 (32.9, 34.1) |
| Variance (8-365 days prior), median (25th, 75th) | 0.4 (0.1, 0.7) | 0.3 (0.2, 0.6) |
| Measurements (8-365 days prior), n, median (25th, 75th) | 1.0 (0.0, 5.0) | 1.0 (0.0, 3.0) |
| **Erythrocyte mean corpuscular hemoglobin, pg** |  |  |
| Minimum (0-7 days prior), median (25th, 75th) | 29.9 (28.3, 31.3) | 30.0 (28.3, 31.4) |
| Maximum (0-7 days prior), median (25th, 75th) | 30.2 (28.6, 31.6) | 30.3 (28.6, 31.7) |
| Average (0-7 days prior), median (25th, 75th) | 30.0 (28.5, 31.4) | 30.1 (28.5, 31.5) |
| Variance (0-7 days prior), median (25th, 75th) | 0.1 (0.0, 0.3) | 0.1 (0.0, 0.2) |
| Measurements (0-7 days prior), n, median (25th, 75th) | 1.0 (0.0, 2.0) | 1.0 (0.0, 2.0) |
| Minimum (8-365 days prior), median (25th, 75th) | 29.4 (27.6, 30.9) | 29.5 (27.7, 31.1) |
| Maximum (8-365 days prior), median (25th, 75th) | 30.5 (28.9, 31.9) | 30.6 (29.0, 32.1) |
| Average (8-365 days prior), median (25th, 75th) | 29.9 (28.3, 31.3) | 30.1 (28.4, 31.5) |
| Variance (8-365 days prior), median (25th, 75th) | 0.3 (0.1, 0.7) | 0.3 (0.1, 0.7) |
| Measurements (8-365 days prior), n, median (25th, 75th) | 1.0 (0.0, 3.0) | 1.0 (0.0, 3.0) |
| **Erythrocyte distribution width, %** |  |  |
| Minimum (0-7 days prior), median (25th, 75th) | 14.1 (13.3, 15.2) | 14.1 (13.3, 15.4) |
| Maximum (0-7 days prior), median (25th, 75th) | 14.4 (13.5, 15.5) | 14.3 (13.5, 15.6) |
| Average (0-7 days prior), median (25th, 75th) | 14.2 (13.4, 15.3) | 14.2 (13.4, 15.5) |
| Variance (0-7 days prior), median (25th, 75th) | 0.1 (0.0, 0.3) | 0.0 (0.0, 0.1) |
| Measurements (0-7 days prior), n, median (25th, 75th) | 1.0 (0.0, 2.0) | 1.0 (0.0, 2.0) |
| Minimum (8-365 days prior), median (25th, 75th) | 13.8 (13.1, 14.7) | 13.8 (13.1, 14.7) |
| Maximum (8-365 days prior), median (25th, 75th) | 14.8 (13.8, 16.3) | 14.6 (13.7, 16.6) |
| Average (8-365 days prior), median (25th, 75th) | 14.3 (13.5, 15.4) | 14.3 (13.5, 15.6) |
| Variance (8-365 days prior), median (25th, 75th) | 0.4 (0.1, 0.9) | 0.2 (0.1, 0.9) |
| Measurements (8-365 days prior), n, median (25th, 75th) | 1.0 (0.0, 3.0) | 1.0 (0.0, 3.0) |
| **Platelets, thou/uL** |  |  |
| Minimum (0-7 days prior), median (25th, 75th) | 223.0 (176.0, 279.0) | 230.0 (181.0, 288.0) |
| Maximum (0-7 days prior), median (25th, 75th) | 241.0 (193.0, 301.0) | 248.0 (198.0, 310.0) |
| Average (0-7 days prior), median (25th, 75th) | 232.0 (185.3, 289.0) | 238.0 (190.0, 297.0) |
| Variance (0-7 days prior), median (25th, 75th) | 420.5 (126.2, 1195.0) | 387.9 (115.0, 1066.3) |
| Measurements (0-7 days prior), n, median (25th, 75th) | 1.0 (0.0, 2.0) | 1.0 (0.0, 2.0) |
| Minimum (8-365 days prior), median (25th, 75th) | 205.0 (157.0, 256.0) | 210.0 (158.0, 263.0) |
| Maximum (8-365 days prior), median (25th, 75th) | 264.0 (209.0, 341.0) | 273.0 (218.0, 351.0) |
| Average (8-365 days prior), median (25th, 75th) | 235.0 (190.0, 289.1) | 241.0 (194.0, 297.0) |
| Variance (8-365 days prior), median (25th, 75th) | 1006.4 (305.1, 3120.5) | 1018.2 (288.0, 3357.0) |
| Measurements (8-365 days prior), n, median (25th, 75th) | 1.0 (0.0, 3.0) | 1.0 (0.0, 4.0) |
| **Platelet mean volume, fL** |  |  |
| Minimum (0-7 days prior), median (25th, 75th) | 8.0 (7.3, 8.7) | 8.2 (7.6, 9.0) |
| Maximum (0-7 days prior), median (25th, 75th) | 8.4 (7.7, 9.2) | 8.5 (7.8, 9.3) |
| Average (0-7 days prior), median (25th, 75th) | 8.2 (7.5, 8.9) | 8.4 (7.7, 9.1) |
| Variance (0-7 days prior), median (25th, 75th) | 0.1 (0.0, 0.4) | 0.0 (0.0, 0.1) |
| Measurements (0-7 days prior), n, median (25th, 75th) | 1.0 (0.0, 2.0) | 1.0 (0.0, 2.0) |
| Minimum (8-365 days prior), median (25th, 75th) | 7.8 (7.1, 8.6) | 8.1 (7.4, 8.9) |
| Maximum (8-365 days prior), median (25th, 75th) | 8.8 (8.1, 9.8) | 9.1 (8.3, 10.1) |
| Average (8-365 days prior), median (25th, 75th) | 8.3 (7.6, 9.0) | 8.5 (7.9, 9.3) |
| Variance (8-365 days prior), median (25th, 75th) | 0.3 (0.1, 0.7) | 0.2 (0.1, 0.6) |
| Measurements (8-365 days prior), n, median (25th, 75th) | 1.0 (0.0, 3.0) | 1.0 (0.0, 3.0) |
| **Neutrophils, thou/uL** |  |  |
| Minimum (0-7 days prior), median (25th, 75th) | 4.0 (2.0, 11.0) | 3.0 (1.0, 7.0) |
| Maximum (0-7 days prior), median (25th, 75th) | 6.0 (2.0, 16.0) | 5.0 (2.0, 11.0) |
| Average (0-7 days prior), median (25th, 75th) | 5.6 (2.0, 13.0) | 4.0 (2.0, 9.0) |
| Variance (0-7 days prior), median (25th, 75th) | 12.5 (2.0, 59.8) | 6.6 (1.0, 36.3) |
| Measurements (0-7 days prior), n, median (25th, 75th) | 0.0 (0.0, 0.0) | 0.0 (0.0, 0.0) |
| Minimum (8-365 days prior), median (25th, 75th) | 2.0 (1.0, 6.0) | 1.0 (1.0, 3.0) |
| Maximum (8-365 days prior), median (25th, 75th) | 8.0 (3.0, 18.0) | 4.0 (2.0, 11.0) |
| Average (8-365 days prior), median (25th, 75th) | 5.0 (2.0, 11.0) | 3.0 (1.7, 6.0) |
| Variance (8-365 days prior), median (25th, 75th) | 21.1 (4.5, 65.7) | 6.2 (1.1, 23.0) |
| Measurements (8-365 days prior), n, median (25th, 75th) | 0.0 (0.0, 0.0) | 0.0 (0.0, 0.0) |
| **Glucose, serum, mg/dL** |  |  |
| Minimum (0-7 days prior), median (25th, 75th) | 100.0 (89.0, 119.0) | 101.0 (89.0, 120.0) |
| Maximum (0-7 days prior), median (25th, 75th) | 118.0 (98.0, 154.0) | 119.0 (100.0, 154.0) |
| Average (0-7 days prior), median (25th, 75th) | 110.0 (96.0, 134.7) | 110.0 (96.1, 135.0) |
| Variance (0-7 days prior), median (25th, 75th) | 8.3 (0.0, 266.7) | 16.0 (0.0, 280.3) |
| Measurements (0-7 days prior), n, median (25th, 75th) | 2.0 (0.0, 4.0) | 2.0 (1.0, 4.0) |
| Minimum (8-365 days prior), median (25th, 75th) | 90.0 (80.0, 102.0) | 91.0 (80.0, 103.0) |
| Maximum (8-365 days prior), median (25th, 75th) | 126.0 (100.0, 175.0) | 129.0 (102.0, 180.0) |
| Average (8-365 days prior), median (25th, 75th) | 107.3 (95.0, 127.0) | 108.1 (96.3, 128.0) |
| Variance (8-365 days prior), median (25th, 75th) | 128.3 (0.0, 571.9) | 144.8 (0.0, 630.4) |
| Measurements (8-365 days prior), n, median (25th, 75th) | 2.0 (0.0, 8.0) | 2.0 (0.0, 8.0) |
| **Urea nitrogen, serum, mg/dL** |  |  |
| Minimum (0-7 days prior), median (25th, 75th) | 14.0 (10.0, 19.0) | 15.0 (11.0, 20.0) |
| Maximum (0-7 days prior), median (25th, 75th) | 16.0 (12.0, 21.0) | 16.0 (12.0, 22.0) |
| Average (0-7 days prior), median (25th, 75th) | 14.7 (11.0, 19.8) | 15.6 (11.9, 21.0) |
| Variance (0-7 days prior), median (25th, 75th) | 0.0 (0.0, 2.7) | 0.0 (0.0, 2.9) |
| Measurements (0-7 days prior), n, median (25th, 75th) | 2.0 (0.0, 4.0) | 2.0 (0.0, 4.0) |
| Minimum (8-365 days prior), median (25th, 75th) | 12.0 (8.0, 16.0) | 13.0 (9.0, 17.0) |
| Maximum (8-365 days prior), median (25th, 75th) | 17.0 (13.0, 24.0) | 19.0 (14.0, 25.0) |
| Average (8-365 days prior), median (25th, 75th) | 14.8 (11.2, 19.0) | 15.5 (12.0, 20.0) |
| Variance (8-365 days prior), median (25th, 75th) | 3.0 (0.0, 12.6) | 3.3 (0.0, 14.4) |
| Measurements (8-365 days prior), n, median (25th, 75th) | 2.0 (0.0, 6.0) | 2.0 (0.0, 7.0) |
| **Creatinine, serum, mg/dL** |  |  |
| Minimum (0-7 days prior), median (25th, 75th) | 0.8 (0.7, 1.0) | 0.8 (0.7, 1.1) |
| Maximum (0-7 days prior), median (25th, 75th) | 0.9 (0.7, 1.1) | 0.9 (0.8, 1.1) |
| Average (0-7 days prior), median (25th, 75th) | 0.9 (0.7, 1.1) | 0.9 (0.7, 1.1) |
| Variance (0-7 days prior), median (25th, 75th) | 0.0 (0.0, 0.0) | 0.0 (0.0, 0.0) |
| Measurements (0-7 days prior), n, median (25th, 75th) | 2.0 (0.0, 4.0) | 2.0 (0.0, 4.0) |
| Minimum (8-365 days prior), median (25th, 75th) | 0.8 (0.6, 1.0) | 0.8 (0.6, 1.0) |
| Maximum (8-365 days prior), median (25th, 75th) | 0.9 (0.8, 1.2) | 1.0 (0.8, 1.2) |
| Average (8-365 days prior), median (25th, 75th) | 0.9 (0.7, 1.1) | 0.9 (0.7, 1.1) |
| Variance (8-365 days prior), median (25th, 75th) | 0.0 (0.0, 0.0) | 0.0 (0.0, 0.0) |
| Measurements (8-365 days prior), n, median (25th, 75th) | 2.0 (0.0, 6.0) | 2.0 (0.0, 8.0) |
| **Sodium, serum, mmol/L** |  |  |
| Minimum (0-7 days prior), median (25th, 75th) | 138.0 (136.0, 140.0) | 138.0 (135.0, 140.0) |
| Maximum (0-7 days prior), median (25th, 75th) | 140.0 (138.0, 141.0) | 139.0 (137.0, 141.0) |
| Average (0-7 days prior), median (25th, 75th) | 139.0 (137.0, 141.0) | 138.5 (136.3, 140.0) |
| Variance (0-7 days prior), median (25th, 75th) | 2.3 (1.0, 6.3) | 2.3 (0.8, 5.4) |
| Measurements (0-7 days prior), n, median (25th, 75th) | 1.0 (0.0, 2.0) | 1.0 (0.0, 2.0) |
| Minimum (8-365 days prior), median (25th, 75th) | 137.0 (134.0, 140.0) | 137.0 (134.0, 139.0) |
| Maximum (8-365 days prior), median (25th, 75th) | 141.0 (139.0, 143.0) | 141.0 (139.0, 142.0) |
| Average (8-365 days prior), median (25th, 75th) | 139.0 (137.3, 140.6) | 139.0 (137.0, 140.2) |
| Variance (8-365 days prior), median (25th, 75th) | 4.3 (2.0, 8.0) | 4.1 (2.0, 7.5) |
| Measurements (8-365 days prior), n, median (25th, 75th) | 1.0 (0.0, 3.0) | 1.0 (0.0, 4.0) |
| **Potassium, serum, mmol/L** |  |  |
| Minimum (0-7 days prior), median (25th, 75th) | 3.9 (3.6, 4.2) | 3.9 (3.6, 4.2) |
| Maximum (0-7 days prior), median (25th, 75th) | 4.2 (3.9, 4.5) | 4.1 (3.9, 4.5) |
| Average (0-7 days prior), median (25th, 75th) | 4.0 (3.8, 4.3) | 4.0 (3.8, 4.3) |
| Variance (0-7 days prior), median (25th, 75th) | 0.1 (0.0, 0.2) | 0.1 (0.0, 0.1) |
| Measurements (0-7 days prior), n, median (25th, 75th) | 1.0 (0.0, 2.0) | 1.0 (0.0, 2.0) |
| Minimum (8-365 days prior), median (25th, 75th) | 3.8 (3.5, 4.1) | 3.8 (3.4, 4.1) |
| Maximum (8-365 days prior), median (25th, 75th) | 4.4 (4.1, 4.8) | 4.4 (4.1, 4.8) |
| Average (8-365 days prior), median (25th, 75th) | 4.1 (3.9, 4.3) | 4.1 (3.9, 4.3) |
| Variance (8-365 days prior), median (25th, 75th) | 0.1 (0.0, 0.2) | 0.1 (0.0, 0.2) |
| Measurements (8-365 days prior), n, median (25th, 75th) | 1.0 (0.0, 3.0) | 1.0 (0.0, 4.0) |
| **Chloride, serum, mmol/L** |  |  |
| Minimum (0-7 days prior), median (25th, 75th) | 101.0 (98.0, 103.0) | 102.0 (100.0, 105.0) |
| Maximum (0-7 days prior), median (25th, 75th) | 103.0 (100.0, 105.0) | 104.0 (102.0, 106.0) |
| Average (0-7 days prior), median (25th, 75th) | 102.0 (99.0, 104.0) | 103.1 (101.0, 105.5) |
| Variance (0-7 days prior), median (25th, 75th) | 4.2 (1.4, 8.3) | 3.5 (1.2, 8.0) |
| Measurements (0-7 days prior), n, median (25th, 75th) | 1.0 (0.0, 2.0) | 1.0 (0.0, 2.0) |
| Minimum (8-365 days prior), median (25th, 75th) | 100.0 (97.0, 102.0) | 101.0 (98.0, 104.0) |
| Maximum (8-365 days prior), median (25th, 75th) | 104.0 (101.0, 107.0) | 105.0 (103.0, 108.0) |
| Average (8-365 days prior), median (25th, 75th) | 102.0 (100.0, 104.0) | 103.5 (101.5, 105.1) |
| Variance (8-365 days prior), median (25th, 75th) | 5.7 (2.3, 11.3) | 4.9 (2.0, 10.3) |
| Measurements (8-365 days prior), n, median (25th, 75th) | 1.0 (0.0, 3.0) | 1.0 (0.0, 4.0) |
| **Carbon dioxide, serum, mmol/L** |  |  |
| Minimum (0-7 days prior), median (25th, 75th) | 24.0 (22.0, 26.0) | 25.0 (22.0, 27.0) |
| Maximum (0-7 days prior), median (25th, 75th) | 26.0 (24.0, 28.0) | 26.0 (24.0, 28.0) |
| Average (0-7 days prior), median (25th, 75th) | 25.0 (23.0, 27.0) | 25.5 (23.5, 27.7) |
| Variance (0-7 days prior), median (25th, 75th) | 2.7 (1.0, 6.3) | 2.9 (1.0, 6.4) |
| Measurements (0-7 days prior), n, median (25th, 75th) | 1.0 (0.0, 2.0) | 1.0 (0.0, 2.0) |
| Minimum (8-365 days prior), median (25th, 75th) | 23.0 (21.0, 26.0) | 24.0 (21.0, 27.0) |
| Maximum (8-365 days prior), median (25th, 75th) | 27.0 (25.0, 29.0) | 28.0 (26.0, 30.0) |
| Average (8-365 days prior), median (25th, 75th) | 25.2 (23.6, 27.0) | 26.0 (24.1, 27.9) |
| Variance (8-365 days prior), median (25th, 75th) | 4.5 (2.0, 8.0) | 4.5 (2.2, 8.0) |
| Measurements (8-365 days prior), n, median (25th, 75th) | 1.0 (0.0, 3.0) | 1.0 (0.0, 4.0) |
| **Lactate, serum, mmol/L** |  |  |
| Minimum (0-7 days prior), median (25th, 75th) | 1.3 (0.9, 1.8) | 1.2 (0.9, 1.8) |
| Maximum (0-7 days prior), median (25th, 75th) | 1.6 (1.1, 2.6) | 1.7 (1.2, 2.6) |
| Average (0-7 days prior), median (25th, 75th) | 1.5 (1.1, 2.2) | 1.5 (1.1, 2.1) |
| Variance (0-7 days prior), median (25th, 75th) | 0.2 (0.0, 0.7) | 0.1 (0.0, 0.5) |
| Measurements (0-7 days prior), n, median (25th, 75th) | 0.0 (0.0, 0.0) | 0.0 (0.0, 0.0) |
| Minimum (8-365 days prior), median (25th, 75th) | 0.9 (0.7, 1.3) | 0.9 (0.7, 1.2) |
| Maximum (8-365 days prior), median (25th, 75th) | 1.7 (1.2, 2.7) | 1.8 (1.2, 2.9) |
| Average (8-365 days prior), median (25th, 75th) | 1.3 (1.0, 1.8) | 1.4 (1.0, 1.8) |
| Variance (8-365 days prior), median (25th, 75th) | 0.3 (0.1, 0.8) | 0.2 (0.1, 0.8) |
| Measurements (8-365 days prior), n, median (25th, 75th) | 0.0 (0.0, 0.0) | 0.0 (0.0, 0.0) |
| **Calcium, serum, mg/dL** |  |  |
| Minimum (0-7 days prior), median (25th, 75th) | 9.1 (8.6, 9.6) | 9.1 (8.6, 9.6) |
| Maximum (0-7 days prior), median (25th, 75th) | 9.4 (9.0, 9.7) | 9.4 (9.0, 9.7) |
| Average (0-7 days prior), median (25th, 75th) | 9.2 (8.8, 9.6) | 9.2 (8.8, 9.6) |
| Variance (0-7 days prior), median (25th, 75th) | 0.0 (0.0, 0.1) | 0.0 (0.0, 0.1) |
| Measurements (0-7 days prior), n, median (25th, 75th) | 2.0 (0.0, 4.0) | 2.0 (0.0, 4.0) |
| Minimum (8-365 days prior), median (25th, 75th) | 9.0 (8.3, 9.5) | 9.0 (8.2, 9.5) |
| Maximum (8-365 days prior), median (25th, 75th) | 9.6 (9.3, 9.9) | 9.6 (9.3, 9.9) |
| Average (8-365 days prior), median (25th, 75th) | 9.3 (8.9, 9.6) | 9.3 (8.9, 9.6) |
| Variance (8-365 days prior), median (25th, 75th) | 0.1 (0.0, 0.2) | 0.1 (0.0, 0.2) |
| Measurements (8-365 days prior), n, median (25th, 75th) | 2.0 (0.0, 6.0) | 2.0 (0.0, 6.0) |
| **Anion gap, serum, mmol/L** |  |  |
| Minimum (0-7 days prior), median (25th, 75th) | 11.0 (9.0, 14.0) | 9.0 (7.0, 10.0) |
| Maximum (0-7 days prior), median (25th, 75th) | 13.0 (11.0, 16.0) | 10.0 (9.0, 13.0) |
| Average (0-7 days prior), median (25th, 75th) | 12.0 (10.0, 14.7) | 9.7 (8.0, 11.0) |
| Variance (0-7 days prior), median (25th, 75th) | 3.7 (1.3, 8.3) | 3.0 (1.1, 8.0) |
| Measurements (0-7 days prior), n, median (25th, 75th) | 1.0 (0.0, 2.0) | 1.0 (0.0, 2.0) |
| Minimum (8-365 days prior), median (25th, 75th) | 10.0 (7.0, 12.0) | 8.0 (6.0, 9.0) |
| Maximum (8-365 days prior), median (25th, 75th) | 14.0 (11.0, 17.0) | 11.0 (9.0, 13.0) |
| Average (8-365 days prior), median (25th, 75th) | 12.0 (9.7, 14.0) | 9.0 (8.0, 10.2) |
| Variance (8-365 days prior), median (25th, 75th) | 4.8 (2.3, 9.3) | 3.5 (2.0, 6.3) |
| Measurements (8-365 days prior), n, median (25th, 75th) | 0.0 (0.0, 3.0) | 1.0 (0.0, 3.0) |
| **Alanine, serum, U/L** |  |  |
| Minimum (0-7 days prior), median (25th, 75th) | 18.0 (12.0, 28.0) | 16.0 (11.0, 26.0) |
| Maximum (0-7 days prior), median (25th, 75th) | 19.0 (12.0, 31.0) | 17.0 (11.0, 28.0) |
| Average (0-7 days prior), median (25th, 75th) | 18.0 (12.0, 30.0) | 16.0 (11.0, 27.0) |
| Variance (0-7 days prior), median (25th, 75th) | 8.0 (2.0, 95.5) | 8.0 (2.0, 60.5) |
| Measurements (0-7 days prior), n, median (25th, 75th) | 0.0 (0.0, 1.0) | 0.0 (0.0, 1.0) |
| Minimum (8-365 days prior), median (25th, 75th) | 15.0 (10.0, 22.0) | 13.0 (9.0, 20.0) |
| Maximum (8-365 days prior), median (25th, 75th) | 21.0 (14.0, 37.0) | 20.0 (13.0, 35.0) |
| Average (8-365 days prior), median (25th, 75th) | 18.5 (13.0, 28.9) | 17.0 (12.0, 26.1) |
| Variance (8-365 days prior), median (25th, 75th) | 26.1 (5.3, 162.0) | 22.7 (4.5, 135.9) |
| Measurements (8-365 days prior), n, median (25th, 75th) | 0.0 (0.0, 1.0) | 0.0 (0.0, 1.0) |
| **Albumin, serum, g/dL** |  |  |
| Minimum (0-7 days prior), median (25th, 75th) | 3.9 (3.3, 4.3) | 3.8 (3.2, 4.2) |
| Maximum (0-7 days prior), median (25th, 75th) | 4.0 (3.4, 4.3) | 3.9 (3.4, 4.3) |
| Average (0-7 days prior), median (25th, 75th) | 3.9 (3.3, 4.3) | 3.9 (3.3, 4.2) |
| Variance (0-7 days prior), median (25th, 75th) | 0.1 (0.0, 0.1) | 0.0 (0.0, 0.1) |
| Measurements (0-7 days prior), n, median (25th, 75th) | 0.0 (0.0, 1.0) | 0.0 (0.0, 1.0) |
| Minimum (8-365 days prior), median (25th, 75th) | 3.9 (3.2, 4.2) | 3.8 (3.2, 4.2) |
| Maximum (8-365 days prior), median (25th, 75th) | 4.2 (3.9, 4.5) | 4.2 (3.9, 4.4) |
| Average (8-365 days prior), median (25th, 75th) | 4.0 (3.6, 4.3) | 4.0 (3.6, 4.3) |
| Variance (8-365 days prior), median (25th, 75th) | 0.1 (0.0, 0.2) | 0.1 (0.0, 0.2) |
| Measurements (8-365 days prior), n, median (25th, 75th) | 0.0 (0.0, 1.0) | 0.0 (0.0, 1.0) |
| **Asparate, serum, U/L** |  |  |
| Minimum (0-7 days prior), median (25th, 75th) | 21.0 (16.0, 30.0) | 21.0 (16.0, 29.0) |
| Maximum (0-7 days prior), median (25th, 75th) | 22.0 (17.0, 34.0) | 22.0 (17.0, 33.0) |
| Average (0-7 days prior), median (25th, 75th) | 22.0 (16.0, 32.0) | 22.0 (17.0, 31.4) |
| Variance (0-7 days prior), median (25th, 75th) | 21.0 (4.5, 145.6) | 18.0 (2.7, 113.5) |
| Measurements (0-7 days prior), n, median (25th, 75th) | 0.0 (0.0, 1.0) | 0.0 (0.0, 1.0) |
| Minimum (8-365 days prior), median (25th, 75th) | 18.0 (14.0, 23.0) | 17.0 (14.0, 22.0) |
| Maximum (8-365 days prior), median (25th, 75th) | 24.0 (18.0, 37.0) | 23.0 (18.0, 37.0) |
| Average (8-365 days prior), median (25th, 75th) | 21.0 (16.1, 29.0) | 20.6 (16.5, 28.0) |
| Variance (8-365 days prior), median (25th, 75th) | 27.0 (6.7, 140.7) | 24.5 (5.0, 133.0) |
| Measurements (8-365 days prior), n, median (25th, 75th) | 0.0 (0.0, 1.0) | 0.0 (0.0, 1.0) |
| **Bilirubin, serum, mg/dL** |  |  |
| Minimum (0-7 days prior), median (25th, 75th) | 0.2 (0.1, 0.2) | 0.1 (0.1, 0.2) |
| Maximum (0-7 days prior), median (25th, 75th) | 0.2 (0.2, 0.2) | 0.1 (0.1, 0.2) |
| Average (0-7 days prior), median (25th, 75th) | 0.2 (0.2, 0.2) | 0.1 (0.1, 0.2) |
| Variance (0-7 days prior), median (25th, 75th) | 0.0 (0.0, 0.0) | 0.0 (0.0, 0.0) |
| Measurements (0-7 days prior), n, median (25th, 75th) | 0.0 (0.0, 0.0) | 0.0 (0.0, 0.0) |
| Minimum (8-365 days prior), median (25th, 75th) | 0.2 (0.1, 0.2) | 0.1 (0.1, 0.2) |
| Maximum (8-365 days prior), median (25th, 75th) | 0.2 (0.2, 0.2) | 0.2 (0.1, 0.3) |
| Average (8-365 days prior), median (25th, 75th) | 0.2 (0.2, 0.2) | 0.1 (0.1, 0.2) |
| Variance (8-365 days prior), median (25th, 75th) | 0.0 (0.0, 0.0) | 0.0 (0.0, 0.0) |
| Measurements (8-365 days prior), n, median (25th, 75th) | 0.0 (0.0, 0.0) | 0.0 (0.0, 0.0) |
| **C-reactive protein, serum, mg/L** |  |  |
| Minimum (0-7 days prior), median (25th, 75th) | 36.1 (6.6, 107.3) | 32.6 (7.0, 110.7) |
| Maximum (0-7 days prior), median (25th, 75th) | 39.9 (7.1, 113.9) | 35.8 (7.4, 117.6) |
| Average (0-7 days prior), median (25th, 75th) | 38.7 (7.0, 111.2) | 35.0 (7.2, 114.3) |
| Variance (0-7 days prior), median (25th, 75th) | 0.0 (0.0, 0.0) | 0.0 (0.0, 0.0) |
| Measurements (0-7 days prior), n, median (25th, 75th) | 0.0 (0.0, 0.0) | 0.0 (0.0, 0.0) |
| Minimum (8-365 days prior), median (25th, 75th) | 9.4 (2.9, 45.4) | 9.7 (2.8, 34.7) |
| Maximum (8-365 days prior), median (25th, 75th) | 33.2 (5.3, 113.3) | 29.4 (6.0, 118.2) |
| Average (8-365 days prior), median (25th, 75th) | 23.3 (4.8, 77.3) | 22.9 (5.3, 73.0) |
| Variance (8-365 days prior), median (25th, 75th) | 0.0 (0.0, 375.2) | 0.0 (0.0, 407.6) |
| Measurements (8-365 days prior), n, median (25th, 75th) | 0.0 (0.0, 0.0) | 0.0 (0.0, 0.0) |
| **Prothrombin time, serum, INR** |  |  |
| Minimum (0-7 days prior), median (25th, 75th) | 1.1 (1.0, 1.2) | 1.0 (1.0, 1.2) |
| Maximum (0-7 days prior), median (25th, 75th) | 1.1 (1.0, 1.2) | 1.1 (1.0, 1.2) |
| Average (0-7 days prior), median (25th, 75th) | 1.1 (1.0, 1.2) | 1.1 (1.0, 1.2) |
| Variance (0-7 days prior), median (25th, 75th) | 0.0 (0.0, 0.0) | 0.0 (0.0, 0.0) |
| Measurements (0-7 days prior), n, median (25th, 75th) | 0.0 (0.0, 1.0) | 0.0 (0.0, 1.0) |
| Minimum (8-365 days prior), median (25th, 75th) | 1.0 (1.0, 1.1) | 1.0 (1.0, 1.1) |
| Maximum (8-365 days prior), median (25th, 75th) | 1.1 (1.0, 1.2) | 1.1 (1.0, 1.3) |
| Average (8-365 days prior), median (25th, 75th) | 1.1 (1.0, 1.2) | 1.1 (1.0, 1.2) |
| Variance (8-365 days prior), median (25th, 75th) | 0.0 (0.0, 0.0) | 0.0 (0.0, 0.0) |
| Measurements (8-365 days prior), n, median (25th, 75th) | 0.0 (0.0, 1.0) | 0.0 (0.0, 1.0) |
| **Erythrocyte sedimentation rate, serum, mm/h** |  |  |
| Minimum (0-7 days prior), median (25th, 75th) | 44.0 (21.0, 80.0) | 11.0 (7.5, 39.0) |
| Maximum (0-7 days prior), median (25th, 75th) | 46.0 (22.0, 84.0) | 11.0 (7.5, 39.0) |
| Average (0-7 days prior), median (25th, 75th) | 45.0 (22.0, 82.0) | 11.0 (7.5, 39.0) |
| Variance (0-7 days prior), median (25th, 75th) | 72.0 (12.5, 378.2) | 0.0 (0.0, 0.0) |
| Measurements (0-7 days prior), n, median (25th, 75th) | 0.0 (0.0, 0.0) | 0.0 (0.0, 0.0) |
| Minimum (8-365 days prior), median (25th, 75th) | 27.0 (11.0, 54.0) | 11.0 (6.0, 28.0) |
| Maximum (8-365 days prior), median (25th, 75th) | 37.0 (17.0, 74.2) | 14.0 (6.0, 31.0) |
| Average (8-365 days prior), median (25th, 75th) | 33.0 (15.0, 64.0) | 14.0 (6.0, 29.0) |
| Variance (8-365 days prior), median (25th, 75th) | 220.5 (34.0, 722.0) | 50.0 (11.7, 331.6) |
| Measurements (8-365 days prior), n, median (25th, 75th) | 0.0 (0.0, 0.0) | 0.0 (0.0, 0.0) |
| **Troponin I, serum, ng/mL** |  |  |
| Minimum (0-7 days prior), median (25th, 75th) | 0.0 (0.0, 0.1) | 0.1 (0.1, 0.1) |
| Maximum (0-7 days prior), median (25th, 75th) | 0.0 (0.0, 0.1) | 0.1 (0.1, 0.1) |
| Average (0-7 days prior), median (25th, 75th) | 0.0 (0.0, 0.1) | 0.1 (0.1, 0.1) |
| Variance (0-7 days prior), median (25th, 75th) | 0.0 (0.0, 0.0) | 0.0 (0.0, 0.0) |
| Measurements (0-7 days prior), n, median (25th, 75th) | 0.0 (0.0, 0.0) | 0.0 (0.0, 0.0) |
| Minimum (8-365 days prior), median (25th, 75th) | 0.0 (0.0, 0.1) | 0.1 (0.1, 0.1) |
| Maximum (8-365 days prior), median (25th, 75th) | 0.0 (0.0, 0.1) | 0.1 (0.1, 0.1) |
| Average (8-365 days prior), median (25th, 75th) | 0.0 (0.0, 0.1) | 0.1 (0.1, 0.1) |
| Variance (8-365 days prior), median (25th, 75th) | 0.0 (0.0, 0.0) | 0.0 (0.0, 0.0) |
| Measurements (8-365 days prior), n, median (25th, 75th) | 0.0 (0.0, 0.0) | 0.0 (0.0, 0.0) |
| **Troponin T, serum, ng/mL** |  |  |
| Minimum (0-7 days prior), median (25th, 75th) | 0.0 (0.0, 0.0) | 0.0 (0.0, 0.0) |
| Maximum (0-7 days prior), median (25th, 75th) | 0.0 (0.0, 0.0) | 0.0 (0.0, 0.0) |
| Average (0-7 days prior), median (25th, 75th) | 0.0 (0.0, 0.0) | 0.0 (0.0, 0.0) |
| Variance (0-7 days prior), median (25th, 75th) | 0.0 (0.0, 0.0) | 0.0 (0.0, 0.0) |
| Measurements (0-7 days prior), n, median (25th, 75th) | 0.0 (0.0, 0.0) | 0.0 (0.0, 0.0) |
| Minimum (8-365 days prior), median (25th, 75th) | 0.0 (0.0, 0.0) | 0.0 (0.0, 0.0) |
| Maximum (8-365 days prior), median (25th, 75th) | 0.0 (0.0, 0.0) | 0.0 (0.0, 0.0) |
| Average (8-365 days prior), median (25th, 75th) | 0.0 (0.0, 0.0) | 0.0 (0.0, 0.0) |
| Variance (8-365 days prior), median (25th, 75th) | 0.0 (0.0, 0.0) | 0.0 (0.0, 0.0) |
| Measurements (8-365 days prior), n, median (25th, 75th) | 0.0 (0.0, 0.0) | 0.0 (0.0, 0.0) |
| **Erythrocytes, urine, /HPF** |  |  |
| Large (0-7 days prior) | 1109 (2.4%) | 435 (2.1%) |
| Moderate (0-7 days prior) | 442 (0.9%) | 256 (1.3%) |
| Small (0-7 days prior) | 1180 (2.5%) | 241 (1.2%) |
| Negative (0-7 days prior) | 6973 (14.8%) | 3325 (16.4%) |
| Missing (0-7 days prior) | 37484 (79.4%) | 16036 (79.0%) |
| Count (0-7 days prior), n, median (25th, 75th) | 0.0 (0.0, 0.0) | 0.0 (0.0, 0.0) |
| Large (8-365 days prior) | 970 (2.1%) | 347 (1.7%) |
| Moderate (8-365 days prior) | 259 (0.5%) | 172 (0.8%) |
| Small (8-365 days prior) | 812 (1.7%) | 129 (0.6%) |
| Negative (8-365 days prior) | 8740 (18.5%) | 3982 (19.6%) |
| Missing (8-365 days prior) | 36407 (77.2%) | 15663 (77.2%) |
| Count (8-365 days prior), n, median (25th, 75th) | 0.0 (0.0, 0.0) | 0.0 (0.0, 0.0) |
| Large (0-365 days prior) | 1674 (3.5%) | 598 (2.9%) |
| Moderate (0-365 days prior) | 527 (1.1%) | 312 (1.5%) |
| Small (0-365 days prior) | 1488 (3.2%) | 284 (1.4%) |
| Negative (0-365 days prior) | 14276 (30.3%) | 6626 (32.7%) |
| Missing (0-365 days prior) | 29223 (61.9%) | 12473 (61.5%) |
| Count (0-365 days prior), n, median (25th, 75th) | 0.0 (0.0, 1.0) | 0.0 (0.0, 2.0) |
| **Protein, urine, mg/dL** |  |  |
| Large (0-365 days prior) | 965 (2.0%) | 418 (2.1%) |
| Moderate (0-365 days prior) | 4937 (10.5%) | 2235 (11.0%) |
| Small (0-365 days prior) | 1281 (2.7%) | 181 (0.9%) |
| Negative (0-365 days prior) | 14757 (31.3%) | 5915 (29.1%) |
| Missing (0-365 days prior) | 25248 (53.5%) | 11544 (56.9%) |
| Count (0-7 days prior), n, median (25th, 75th) | 0.0 (0.0, 0.0) | 0.0 (0.0, 0.0) |
| Count (8-365 days prior), n, median (25th, 75th) | 0.0 (0.0, 1.0) | 0.0 (0.0, 1.0) |
| Count (0-365 days prior), n, median (25th, 75th) | 0.0 (0.0, 1.0) | 0.0 (0.0, 1.0) |
| **Glucose, urine, mg/dL** |  |  |
| Large (0-7 days prior) | 242 (0.5%) | 19 (0.1%) |
| Moderate (0-7 days prior) | 345 (0.7%) | 254 (1.3%) |
| Small (0-7 days prior) | 649 (1.4%) | 270 (1.3%) |
| Negative (0-7 days prior) | 9893 (21.0%) | 3887 (19.2%) |
| Missing (0-7 days prior) | 36059 (76.4%) | 15863 (78.2%) |
| Count (0-7 days prior), n, median (25th, 75th) | 0.0 (0.0, 0.0) | 0.0 (0.0, 0.0) |
| Large (8-365 days prior) | 325 (0.7%) | 58 (0.3%) |
| Moderate (8-365 days prior) | 289 (0.6%) | 226 (1.1%) |
| Small (8-365 days prior) | 367 (0.8%) | 146 (0.7%) |
| Negative (8-365 days prior) | 13289 (28.2%) | 5116 (25.2%) |
| Missing (8-365 days prior) | 32918 (69.8%) | 14747 (72.7%) |
| Count (8-365 days prior), n, median (25th, 75th) | 0.0 (0.0, 1.0) | 0.0 (0.0, 1.0) |
| **Hemoglobin, urine, /HPF** |  |  |
| Count (0-7 days prior), n, median (25th, 75th) | 0.0 (0.0, 0.0) | 0.0 (0.0, 0.0) |
| Count (8-365 days prior), n, median (25th, 75th) | 0.0 (0.0, 0.0) | 0.0 (0.0, 0.0) |

**Supplementary Table S4. Summary of intraoperative physiological variables.**

|  | **Development Cohort** (6/1/2014 - 11/26/2018) | **Validation Cohort** (11/27/2018 - 9/20/2020) |
| --- | --- | --- |
| **Patients, n** | 38621 | 17621 |
| **Hospital encounters, n** | 47188 | 20293 |
| **Systolic blood pressure** |  |  |
| Measured value, mmHg, median (25th, 75th) | 113.0 (100.0, 128.0) | 114.0 (101.0, 130.0) |
| Total measurements, n, (25th, 75th) | 66.0 (34.0, 138.0) | 67.0 (29.0, 157.0) |
| Frequency, n/hour, (25th, 75th) | 20.1 (17.7, 46.3) | 20.0 (17.1, 48.9) |
| Encounters missing, n (%) | 36 (0.1%) | 14 (0.1%) |
| **Diastolic blood pressure** |  |  |
| Measured value, mmHg, median (25th, 75th) | 62.0 (53.2, 71.0) | 63.0 (54.0, 73.0) |
| Total measurements, n, (25th, 75th) | 66.0 (34.0, 138.0) | 67.0 (29.0, 157.0) |
| Frequency, n/hour, (25th, 75th) | 20.1 (17.7, 46.3) | 20.0 (17.1, 48.9) |
| Encounters missing, n (%) | 36 (0.1%) | 14 (0.1%) |
| **Mean arterial pressure** |  |  |
| Measured value, mmHg, median (25th, 75th) | 78.0 (69.0, 89.0) | 80.5 (71.0, 92.0) |
| Total measurements, n, (25th, 75th) | 66.0 (34.0, 139.0) | 67.0 (29.0, 158.0) |
| Frequency, n/hour, (25th, 75th) | 20.1 (17.7, 46.6) | 20.0 (17.1, 49.2) |
| Encounters missing, n (%) | 36 (0.1%) | 14 (0.1%) |
| **Heart rate** |  |  |
| Measured value, bpm, median (25th, 75th) | 75.5 (65.5, 87.0) | 75.5 (65.5, 87.0) |
| Total measurements, n, (25th, 75th) | 161.0 (88.0, 247.0) | 157.0 (75.0, 245.0) |
| Frequency, n/hour, (25th, 75th) | 54.9 (49.8, 56.9) | 54.1 (46.8, 56.4) |
| Encounters missing, n (%) | 38 (0.1%) | 10 (0.0%) |
| **Oxygen saturation (SpO2)** |  |  |
| Measured value, %, median (25th, 75th) | 99.0 (97.4, 100.0) | 99.0 (97.1, 100.0) |
| Total measurements, n, (25th, 75th) | 162.0 (86.0, 254.0) | 159.0 (73.0, 258.0) |
| Frequency, n/hour, (25th, 75th) | 54.5 (47.5, 56.9) | 53.7 (43.1, 56.6) |
| Encounters missing, n (%) | 27 (0.1%) | 8 (0.0%) |
| **Fraction of inspired oxygen (FiO2)** |  |  |
| Measured value, %, median (25th, 75th) | 40.0 (40.0, 40.0) | 40.0 (40.0, 40.0) |
| Total measurements, n, (25th, 75th) | 171.0 (95.0, 271.0) | 171.0 (83.0, 276.0) |
| Frequency, n/hour, (25th, 75th) | 57.1 (53.5, 58.6) | 56.5 (51.8, 58.5) |
| Encounters missing, n (%) | 27 (0.1%) | 8 (0.0%) |
| **End-tidal carbon dioxide (EtCO2)** |  |  |
| Measured value, mmHg, median (25th, 75th) | 34.0 (32.0, 37.0) | 35.0 (32.0, 38.0) |
| Total measurements, n, (25th, 75th) | 130.0 (2.0, 224.0) | 0.0 (0.0, 152.0) |
| Frequency, n/hour, (25th, 75th) | 49.1 (0.5, 53.5) | 0.0 (0.0, 47.9) |
| Encounters missing, n (%) | 27 (0.1%) | 8 (0.0%) |
| **Tidal volume** |  |  |
| Measured value, mL, median (25th, 75th) | 442.0 (361.0, 508.0) | 467.0 (388.0, 527.0) |
| Total measurements, n, (25th, 75th) | 154.0 (78.0, 248.0) | 136.0 (0.0, 238.0) |
| Frequency, n/hour, (25th, 75th) | 51.9 (43.5, 55.2) | 49.6 (0.0, 54.3) |
| Encounters missing, n (%) | 27 (0.1%) | 8 (0.0%) |
| **Respiration rate** |  |  |
| Measured value, breaths/minute, median (25th, 75th) | 10.0 (8.0, 12.0) | 12.0 (10.0, 14.0) |
| Total measurements, n, (25th, 75th) | 167.0 (91.0, 265.0) | 63.0 (1.0, 185.0) |
| Frequency, n/hour, (25th, 75th) | 55.9 (51.1, 58.0) | 43.4 (0.2, 54.6) |
| Encounters missing, n (%) | 27 (0.1%) | 8 (0.0%) |
| **Peak inspiratory pressure** |  |  |
| Measured value, mmHg, median (25th, 75th) | 18.0 (13.0, 23.0) | 18.0 (13.0, 22.0) |
| Total measurements, n, (25th, 75th) | 168.0 (91.0, 266.0) | 153.0 (43.0, 259.0) |
| Frequency, n/hour, (25th, 75th) | 56.1 (51.8, 58.0) | 54.8 (36.5, 57.6) |
| Encounters missing, n (%) | 27 (0.1%) | 8 (0.0%) |
| **Minimum alveolar concentration** |  |  |
| Measured value, median (25th, 75th) | 0.6 (0.4, 0.8) | 0.5 (0.3, 0.8) |
| Total measurements, n, (25th, 75th) | 140.0 (67.0, 227.0) | 150.0 (67.0, 238.0) |
| Frequency, n/hour, (25th, 75th) | 48.8 (39.0, 53.1) | 52.6 (42.9, 56.0) |
| Encounters missing, n (%) | 4868 (10.3%) | 2192 (10.8%) |
| **Core temperature** |  |  |
| Measured value, degrees Celsius, median (25th, 75th) | 36.8 (36.2, 37.3) | 36.8 (36.3, 37.4) |
| Total measurements, n, (25th, 75th) | 106.0 (22.0, 190.2) | 76.0 (1.0, 169.0) |
| Frequency, n/hour, (25th, 75th) | 38.8 (14.6, 46.3) | 32.7 (0.3, 44.0) |
| Encounters missing, n (%) | 3025 (6.4%) | 1758 (8.7%) |
| **Urine output sum, mL, median (25th, 75th)** | 160.0 (0.0, 500.0) | 100.0 (0.0, 515.0) |
| **Blood loss sum, mL, median (25th, 75th)** | 50.0 (0.0, 200.0) | 25.0 (0.0, 200.0) |

**Supplementary Table S5. Classification performance metrics for all experimental models and prediction phases in validation cohort.**

|  |  |  |  |  |  |  |  |
| --- | --- | --- | --- | --- | --- | --- | --- |
|  | **Sensitivity (95% CI)** | **Specificity (95% CI)** | **PPV (95% CI)** | **NPV (95% CI)** | **Accuracy (95% CI)** | **AUPRC (95% CI)** | **AUROC (95% CI)** |
| **Prolonged ICU Stay** | | | | | | | |
| Random Forest Preop | 0.81 (0.77-0.83) | 0.77 (0.75-0.81) | 0.64 (0.62-0.67) | 0.89 (0.87-0.90) | 0.78 (0.77-0.80) | 0.78 (0.76-0.79) | 0.87 (0.87-0.88) |
| XGBoost Preop | 0.81 (0.78-0.83) | 0.80 (0.78-0.82) | 0.67 (0.65-0.69) | 0.89 (0.88-0.90) | 0.80 (0.79-0.81) | 0.81 (0.80-0.82) | 0.89 (0.88-0.89) |
| Deep Preop | 0.80 (0.78-0.83) | 0.80 (0.77-0.82) | 0.67 (0.64-0.69) | 0.89 (0.88-0.90) | 0.80 (0.79-0.81) | 0.82 (0.81-0.82) | 0.89 (0.88-0.89) |
| Deep Preop (Multi-Task) | 0.81 (0.79-0.83) | 0.78 (0.76-0.80) | 0.65 (0.63-0.67) | 0.89 (0.88-0.90) | 0.79 (0.78-0.80) | 0.80 (0.79-0.81) | 0.88 (0.88-0.89) |
| Random Forest Intraop | 0.79 (0.77-0.83) | 0.80 (0.76-0.82) | 0.66 (0.63-0.68) | 0.88 (0.88-0.90) | 0.80 (0.78-0.80) | 0.80 (0.79-0.81) | 0.88 (0.87-0.88) |
| XGBoost Intraop | 0.79 (0.74-0.81) | 0.82 (0.80-0.87) | 0.69 (0.67-0.74) | 0.89 (0.87-0.89) | 0.81 (0.80-0.83) | 0.83 (0.82-0.84) | 0.88 (0.88-0.89) |
| Deep Intraop | 0.81 (0.79-0.83) | 0.82 (0.81-0.84) | 0.69 (0.68-0.72) | 0.90 (0.89-0.90) | 0.82 (0.81-0.83) | 0.83 (0.82-0.84) | 0.89 (0.88-0.89) |
| Deep Intraop (Multi-Task) | 0.77 (0.74-0.80) | 0.84 (0.82-0.87) | 0.71 (0.68-0.74) | 0.88 (0.87-0.89) | 0.82 (0.81-0.83) | 0.83 (0.82-0.84) | 0.88 (0.88-0.89) |
| Random Forest Postop | 0.80 (0.78-0.82) | 0.83 (0.81-0.86) | 0.71 (0.69-0.73) | 0.89 (0.89-0.90) | 0.82 (0.82-0.83) | 0.83 (0.83-0.84) | 0.90 (0.89-0.90) |
| XGBoost Postop | 0.84 (0.83-0.85) | 0.84 (0.83-0.86) | 0.73 (0.71-0.74) | 0.91 (0.91-0.92) | 0.84 (0.83-0.85) | 0.87 (0.86-0.87) | 0.92 (0.91-0.92) |
| Deep Postop | 0.83 (0.80-0.85) | 0.84 (0.83-0.87) | 0.72 (0.71-0.76) | 0.91 (0.90-0.92) | 0.84 (0.83-0.85) | 0.87 (0.86-0.88) | 0.92 (0.91-0.92) |
| Deep Postop (Multi-Task) | 0.83 (0.81-0.86) | 0.83 (0.80-0.85) | 0.71 (0.68-0.73) | 0.91 (0.90-0.92) | 0.83 (0.82-0.84) | 0.86 (0.85-0.86) | 0.91 (0.91-0.92) |
| **Prolonged Mechanical Ventilation** | | | | | | | |
| Random Forest Preop | 0.77 (0.75-0.81) | 0.79 (0.75-0.80) | 0.24 (0.21-0.25) | 0.98 (0.97-0.98) | 0.79 (0.76-0.80) | 0.46 (0.44-0.49) | 0.86 (0.85-0.87) |
| XGBoost Preop | 0.76 (0.75-0.83) | 0.83 (0.78-0.84) | 0.28 (0.23-0.30) | 0.98 (0.98-0.98) | 0.83 (0.78-0.84) | 0.50 (0.47-0.53) | 0.88 (0.87-0.89) |
| Deep Preop | 0.77 (0.75-0.84) | 0.85 (0.78-0.87) | 0.30 (0.24-0.32) | 0.98 (0.98-0.98) | 0.84 (0.79-0.86) | 0.52 (0.50-0.55) | 0.89 (0.88-0.90) |
| Deep Preop (Multi-Task) | 0.84 (0.80-0.87) | 0.80 (0.76-0.84) | 0.26 (0.23-0.29) | 0.98 (0.98-0.99) | 0.80 (0.77-0.84) | 0.53 (0.50-0.55) | 0.90 (0.89-0.90) |
| Random Forest Intraop | 0.81 (0.77-0.85) | 0.85 (0.81-0.88) | 0.32 (0.27-0.36) | 0.98 (0.98-0.99) | 0.85 (0.82-0.88) | 0.55 (0.52-0.58) | 0.90 (0.89-0.91) |
| XGBoost Intraop | 0.79 (0.76-0.85) | 0.89 (0.82-0.90) | 0.37 (0.28-0.41) | 0.98 (0.98-0.99) | 0.88 (0.82-0.89) | 0.58 (0.55-0.61) | 0.90 (0.89-0.91) |
| Deep Intraop | 0.84 (0.82-0.86) | 0.85 (0.83-0.86) | 0.32 (0.30-0.34) | 0.98 (0.98-0.99) | 0.85 (0.84-0.86) | 0.59 (0.57-0.62) | 0.92 (0.91-0.92) |
| Deep Intraop (Multi-Task) | 0.85 (0.80-0.86) | 0.84 (0.84-0.89) | 0.31 (0.30-0.38) | 0.99 (0.98-0.99) | 0.84 (0.84-0.88) | 0.59 (0.56-0.61) | 0.91 (0.91-0.92) |
| Random Forest Postop | 0.82 (0.79-0.87) | 0.86 (0.81-0.88) | 0.33 (0.28-0.37) | 0.98 (0.98-0.99) | 0.86 (0.81-0.88) | 0.58 (0.55-0.60) | 0.91 (0.91-0.92) |
| XGBoost Postop | 0.85 (0.81-0.87) | 0.86 (0.84-0.89) | 0.33 (0.31-0.39) | 0.99 (0.98-0.99) | 0.86 (0.84-0.88) | 0.62 (0.59-0.64) | 0.92 (0.91-0.93) |
| Deep Postop | 0.84 (0.82-0.89) | 0.85 (0.80-0.87) | 0.31 (0.27-0.35) | 0.98 (0.98-0.99) | 0.85 (0.80-0.86) | 0.61 (0.59-0.64) | 0.92 (0.92-0.93) |
| Deep Postop (Multi-Task) | 0.87 (0.83-0.88) | 0.84 (0.84-0.88) | 0.32 (0.31-0.37) | 0.99 (0.98-0.99) | 0.85 (0.84-0.88) | 0.62 (0.59-0.65) | 0.93 (0.92-0.94) |
| **Wound Complications** | | | | | | | |
| Random Forest Preop | 0.65 (0.59-0.69) | 0.68 (0.64-0.75) | 0.36 (0.34-0.38) | 0.88 (0.87-0.88) | 0.67 (0.65-0.71) | 0.45 (0.43-0.47) | 0.73 (0.72-0.73) |
| XGBoost Preop | 0.69 (0.62-0.71) | 0.68 (0.67-0.76) | 0.37 (0.36-0.41) | 0.89 (0.88-0.90) | 0.69 (0.68-0.73) | 0.52 (0.50-0.54) | 0.76 (0.75-0.77) |
| Deep Preop | 0.62 (0.60-0.75) | 0.76 (0.64-0.78) | 0.41 (0.36-0.43) | 0.88 (0.88-0.90) | 0.73 (0.66-0.74) | 0.52 (0.50-0.53) | 0.76 (0.75-0.77) |
| Deep Preop (Multi-Task) | 0.67 (0.65-0.77) | 0.72 (0.61-0.73) | 0.39 (0.35-0.41) | 0.89 (0.88-0.91) | 0.71 (0.65-0.72) | 0.53 (0.51-0.54) | 0.77 (0.76-0.78) |
| Random Forest Intraop | 0.56 (0.49-0.73) | 0.55 (0.40-0.63) | 0.26 (0.24-0.27) | 0.82 (0.82-0.85) | 0.56 (0.47-0.60) | 0.27 (0.26-0.28) | 0.59 (0.58-0.60) |
| XGBoost Intraop | 0.39 (0.35-0.63) | 0.72 (0.49-0.77) | 0.28 (0.25-0.29) | 0.81 (0.81-0.83) | 0.65 (0.52-0.68) | 0.27 (0.26-0.28) | 0.57 (0.56-0.58) |
| Deep Intraop | 0.40 (0.36-0.59) | 0.76 (0.57-0.81) | 0.31 (0.27-0.33) | 0.82 (0.82-0.84) | 0.68 (0.58-0.71) | 0.31 (0.30-0.33) | 0.61 (0.60-0.62) |
| Deep Intraop (Multi-Task) | 0.45 (0.38-0.61) | 0.74 (0.57-0.80) | 0.31 (0.28-0.35) | 0.83 (0.82-0.84) | 0.67 (0.58-0.71) | 0.33 (0.32-0.34) | 0.62 (0.61-0.63) |
| Random Forest Postop | 0.63 (0.62-0.73) | 0.68 (0.57-0.68) | 0.35 (0.32-0.36) | 0.87 (0.87-0.89) | 0.67 (0.61-0.67) | 0.42 (0.40-0.43) | 0.71 (0.70-0.72) |
| XGBoost Postop | 0.63 (0.61-0.73) | 0.73 (0.64-0.75) | 0.39 (0.35-0.41) | 0.88 (0.88-0.90) | 0.71 (0.66-0.72) | 0.50 (0.48-0.51) | 0.75 (0.74-0.76) |
| Deep Postop | 0.69 (0.64-0.73) | 0.72 (0.68-0.77) | 0.40 (0.38-0.43) | 0.89 (0.89-0.90) | 0.71 (0.69-0.74) | 0.53 (0.52-0.55) | 0.78 (0.77-0.78) |
| Deep Postop (Multi-Task) | 0.74 (0.68-0.76) | 0.66 (0.65-0.72) | 0.38 (0.36-0.41) | 0.90 (0.89-0.91) | 0.68 (0.67-0.72) | 0.52 (0.51-0.54) | 0.77 (0.77-0.78) |
| **Neurological Complications** | | | | | | | |
| Random Forest Preop | 0.79 (0.73-0.82) | 0.70 (0.67-0.76) | 0.40 (0.39-0.44) | 0.93 (0.92-0.94) | 0.72 (0.70-0.75) | 0.59 (0.57-0.60) | 0.83 (0.82-0.83) |
| XGBoost Preop | 0.78 (0.74-0.81) | 0.74 (0.72-0.78) | 0.43 (0.42-0.46) | 0.93 (0.92-0.94) | 0.75 (0.73-0.77) | 0.62 (0.60-0.64) | 0.84 (0.83-0.85) |
| Deep Preop | 0.77 (0.73-0.83) | 0.77 (0.71-0.81) | 0.46 (0.42-0.49) | 0.93 (0.92-0.94) | 0.77 (0.73-0.79) | 0.63 (0.62-0.65) | 0.85 (0.84-0.86) |
| Deep Preop (Multi-Task) | 0.79 (0.78-0.81) | 0.74 (0.73-0.76) | 0.44 (0.42-0.45) | 0.93 (0.93-0.94) | 0.75 (0.74-0.76) | 0.63 (0.61-0.64) | 0.85 (0.84-0.85) |
| Random Forest Intraop | 0.76 (0.68-0.77) | 0.61 (0.60-0.69) | 0.33 (0.32-0.36) | 0.91 (0.89-0.91) | 0.64 (0.63-0.69) | 0.45 (0.44-0.47) | 0.75 (0.74-0.76) |
| XGBoost Intraop | 0.64 (0.59-0.72) | 0.73 (0.65-0.77) | 0.37 (0.34-0.40) | 0.89 (0.88-0.90) | 0.71 (0.66-0.74) | 0.47 (0.46-0.49) | 0.75 (0.74-0.76) |
| Deep Intraop | 0.70 (0.65-0.73) | 0.67 (0.65-0.72) | 0.35 (0.34-0.37) | 0.90 (0.89-0.91) | 0.68 (0.66-0.70) | 0.45 (0.44-0.47) | 0.75 (0.74-0.76) |
| Deep Intraop (Multi-Task) | 0.71 (0.63-0.75) | 0.67 (0.63-0.75) | 0.35 (0.34-0.39) | 0.90 (0.89-0.91) | 0.68 (0.65-0.73) | 0.48 (0.46-0.50) | 0.76 (0.75-0.77) |
| Random Forest Postop | 0.76 (0.70-0.82) | 0.70 (0.64-0.77) | 0.39 (0.37-0.43) | 0.92 (0.91-0.93) | 0.71 (0.68-0.75) | 0.54 (0.52-0.55) | 0.81 (0.80-0.81) |
| XGBoost Postop | 0.78 (0.72-0.82) | 0.74 (0.71-0.80) | 0.43 (0.41-0.48) | 0.93 (0.92-0.94) | 0.75 (0.73-0.78) | 0.62 (0.60-0.63) | 0.84 (0.83-0.85) |
| Deep Postop | 0.80 (0.75-0.82) | 0.75 (0.73-0.80) | 0.45 (0.43-0.49) | 0.94 (0.93-0.94) | 0.76 (0.75-0.79) | 0.64 (0.62-0.65) | 0.86 (0.85-0.86) |
| Deep Postop (Multi-Task) | 0.83 (0.76-0.84) | 0.71 (0.70-0.78) | 0.42 (0.41-0.47) | 0.94 (0.93-0.95) | 0.74 (0.73-0.77) | 0.64 (0.62-0.65) | 0.85 (0.85-0.86) |
| **Cardiovascular Complications** | | | | | | | |
| Random Forest Preop | 0.74 (0.70-0.78) | 0.69 (0.65-0.73) | 0.32 (0.30-0.33) | 0.93 (0.93-0.94) | 0.70 (0.67-0.72) | 0.42 (0.40-0.44) | 0.78 (0.77-0.79) |
| XGBoost Preop | 0.72 (0.67-0.76) | 0.71 (0.67-0.76) | 0.32 (0.31-0.35) | 0.93 (0.92-0.94) | 0.71 (0.69-0.74) | 0.43 (0.42-0.45) | 0.79 (0.78-0.79) |
| Deep Preop | 0.72 (0.67-0.77) | 0.75 (0.70-0.80) | 0.36 (0.33-0.39) | 0.93 (0.93-0.94) | 0.74 (0.71-0.78) | 0.48 (0.46-0.50) | 0.81 (0.80-0.82) |
| Deep Preop (Multi-Task) | 0.72 (0.67-0.75) | 0.74 (0.71-0.78) | 0.35 (0.33-0.38) | 0.93 (0.92-0.94) | 0.74 (0.72-0.76) | 0.48 (0.46-0.50) | 0.81 (0.80-0.81) |
| Random Forest Intraop | 0.72 (0.71-0.77) | 0.76 (0.71-0.76) | 0.37 (0.33-0.38) | 0.93 (0.93-0.94) | 0.75 (0.72-0.76) | 0.46 (0.45-0.48) | 0.81 (0.80-0.82) |
| XGBoost Intraop | 0.75 (0.68-0.78) | 0.74 (0.71-0.81) | 0.36 (0.34-0.41) | 0.94 (0.93-0.94) | 0.74 (0.72-0.79) | 0.51 (0.49-0.52) | 0.82 (0.81-0.82) |
| Deep Intraop | 0.74 (0.71-0.78) | 0.78 (0.73-0.80) | 0.39 (0.36-0.42) | 0.94 (0.93-0.95) | 0.77 (0.74-0.79) | 0.53 (0.51-0.55) | 0.83 (0.82-0.84) |
| Deep Intraop (Multi-Task) | 0.76 (0.72-0.77) | 0.76 (0.76-0.80) | 0.38 (0.37-0.41) | 0.94 (0.94-0.95) | 0.76 (0.76-0.79) | 0.53 (0.51-0.55) | 0.83 (0.82-0.84) |
| Random Forest Postop | 0.74 (0.73-0.81) | 0.77 (0.70-0.77) | 0.38 (0.34-0.39) | 0.94 (0.94-0.95) | 0.76 (0.72-0.77) | 0.48 (0.46-0.50) | 0.82 (0.81-0.83) |
| XGBoost Postop | 0.76 (0.71-0.79) | 0.76 (0.72-0.81) | 0.38 (0.35-0.42) | 0.94 (0.93-0.95) | 0.76 (0.73-0.79) | 0.52 (0.50-0.54) | 0.83 (0.82-0.84) |
| Deep Postop | 0.77 (0.74-0.82) | 0.77 (0.72-0.81) | 0.40 (0.36-0.43) | 0.95 (0.94-0.96) | 0.77 (0.74-0.80) | 0.56 (0.54-0.58) | 0.85 (0.84-0.86) |
| Deep Postop (Multi-Task) | 0.83 (0.77-0.84) | 0.72 (0.71-0.78) | 0.37 (0.36-0.40) | 0.96 (0.95-0.96) | 0.74 (0.73-0.78) | 0.56 (0.54-0.58) | 0.85 (0.85-0.86) |
| **Sepsis** | | | | | | | |
| Random Forest Preop | 0.73 (0.68-0.77) | 0.81 (0.77-0.85) | 0.27 (0.24-0.32) | 0.97 (0.97-0.97) | 0.81 (0.77-0.84) | 0.45 (0.43-0.48) | 0.85 (0.84-0.86) |
| XGBoost Preop | 0.76 (0.70-0.79) | 0.78 (0.75-0.84) | 0.25 (0.23-0.29) | 0.97 (0.97-0.97) | 0.78 (0.75-0.83) | 0.45 (0.43-0.48) | 0.85 (0.84-0.86) |
| Deep Preop | 0.79 (0.73-0.82) | 0.78 (0.76-0.83) | 0.26 (0.24-0.30) | 0.97 (0.97-0.98) | 0.78 (0.76-0.82) | 0.48 (0.46-0.50) | 0.86 (0.86-0.87) |
| Deep Preop (Multi-Task) | 0.80 (0.75-0.83) | 0.78 (0.74-0.83) | 0.26 (0.24-0.29) | 0.98 (0.97-0.98) | 0.78 (0.75-0.82) | 0.48 (0.45-0.50) | 0.87 (0.86-0.88) |
| Random Forest Intraop | 0.70 (0.66-0.72) | 0.72 (0.71-0.73) | 0.19 (0.18-0.21) | 0.96 (0.96-0.96) | 0.72 (0.71-0.73) | 0.30 (0.28-0.32) | 0.77 (0.75-0.78) |
| XGBoost Intraop | 0.64 (0.55-0.73) | 0.75 (0.67-0.84) | 0.20 (0.17-0.25) | 0.96 (0.95-0.96) | 0.74 (0.68-0.81) | 0.31 (0.28-0.33) | 0.77 (0.75-0.78) |
| Deep Intraop | 0.74 (0.67-0.76) | 0.68 (0.67-0.74) | 0.18 (0.17-0.21) | 0.96 (0.96-0.97) | 0.69 (0.68-0.74) | 0.32 (0.30-0.34) | 0.78 (0.77-0.79) |
| Deep Intraop (Multi-Task) | 0.70 (0.65-0.75) | 0.76 (0.72-0.81) | 0.22 (0.20-0.25) | 0.96 (0.96-0.97) | 0.75 (0.72-0.80) | 0.34 (0.32-0.37) | 0.80 (0.78-0.81) |
| Random Forest Postop | 0.75 (0.72-0.79) | 0.80 (0.76-0.83) | 0.26 (0.23-0.28) | 0.97 (0.97-0.97) | 0.79 (0.76-0.82) | 0.41 (0.38-0.43) | 0.85 (0.84-0.85) |
| XGBoost Postop | 0.78 (0.72-0.80) | 0.78 (0.77-0.84) | 0.25 (0.24-0.30) | 0.97 (0.97-0.98) | 0.78 (0.77-0.83) | 0.46 (0.43-0.48) | 0.86 (0.85-0.87) |
| Deep Postop | 0.80 (0.72-0.82) | 0.79 (0.78-0.87) | 0.27 (0.25-0.35) | 0.98 (0.97-0.98) | 0.79 (0.78-0.86) | 0.50 (0.47-0.52) | 0.87 (0.87-0.88) |
| Deep Postop (Multi-Task) | 0.78 (0.75-0.82) | 0.81 (0.78-0.84) | 0.29 (0.25-0.32) | 0.97 (0.97-0.98) | 0.81 (0.78-0.83) | 0.50 (0.48-0.52) | 0.88 (0.87-0.88) |
| **Acute Kidney Injury** | | | | | | | |
| Random Forest Preop | 0.75 (0.71-0.81) | 0.70 (0.64-0.73) | 0.34 (0.31-0.36) | 0.93 (0.93-0.94) | 0.71 (0.67-0.73) | 0.45 (0.43-0.47) | 0.80 (0.79-0.80) |
| XGBoost Preop | 0.76 (0.73-0.84) | 0.72 (0.64-0.75) | 0.36 (0.32-0.37) | 0.94 (0.93-0.95) | 0.73 (0.67-0.75) | 0.51 (0.49-0.53) | 0.82 (0.81-0.83) |
| Deep Preop | 0.78 (0.75-0.79) | 0.71 (0.70-0.74) | 0.35 (0.34-0.37) | 0.94 (0.93-0.94) | 0.72 (0.71-0.74) | 0.51 (0.49-0.53) | 0.82 (0.81-0.82) |
| Deep Preop (Multi-Task) | 0.74 (0.72-0.79) | 0.74 (0.69-0.76) | 0.37 (0.34-0.39) | 0.93 (0.93-0.94) | 0.74 (0.71-0.76) | 0.51 (0.49-0.53) | 0.82 (0.81-0.83) |
| Random Forest Intraop | 0.55 (0.53-0.61) | 0.76 (0.71-0.76) | 0.32 (0.29-0.33) | 0.89 (0.89-0.90) | 0.72 (0.69-0.73) | 0.36 (0.35-0.38) | 0.71 (0.70-0.72) |
| XGBoost Intraop | 0.59 (0.56-0.61) | 0.74 (0.72-0.76) | 0.31 (0.30-0.33) | 0.90 (0.89-0.90) | 0.71 (0.70-0.73) | 0.38 (0.37-0.40) | 0.71 (0.70-0.72) |
| Deep Intraop | 0.67 (0.53-0.71) | 0.65 (0.62-0.79) | 0.28 (0.27-0.35) | 0.91 (0.89-0.91) | 0.66 (0.63-0.75) | 0.40 (0.38-0.42) | 0.73 (0.72-0.74) |
| Deep Intraop (Multi-Task) | 0.57 (0.50-0.67) | 0.77 (0.67-0.84) | 0.33 (0.29-0.38) | 0.90 (0.89-0.91) | 0.73 (0.67-0.78) | 0.41 (0.39-0.43) | 0.74 (0.73-0.74) |
| Random Forest Postop | 0.73 (0.72-0.79) | 0.73 (0.67-0.74) | 0.36 (0.33-0.37) | 0.93 (0.93-0.94) | 0.73 (0.69-0.74) | 0.45 (0.43-0.47) | 0.80 (0.79-0.81) |
| XGBoost Postop | 0.78 (0.70-0.80) | 0.70 (0.69-0.78) | 0.35 (0.34-0.40) | 0.94 (0.93-0.94) | 0.71 (0.71-0.77) | 0.50 (0.48-0.52) | 0.81 (0.81-0.82) |
| Deep Postop | 0.74 (0.72-0.82) | 0.76 (0.68-0.78) | 0.39 (0.34-0.40) | 0.93 (0.93-0.95) | 0.76 (0.70-0.77) | 0.54 (0.52-0.55) | 0.83 (0.82-0.83) |
| Deep Postop (Multi-Task) | 0.74 (0.70-0.82) | 0.76 (0.68-0.79) | 0.38 (0.34-0.41) | 0.93 (0.93-0.95) | 0.75 (0.70-0.78) | 0.52 (0.51-0.54) | 0.82 (0.82-0.83) |
| **Venous Thromboembolism** | | | | | | | |
| Random Forest Preop | 0.69 (0.60-0.77) | 0.73 (0.66-0.82) | 0.13 (0.11-0.16) | 0.98 (0.97-0.98) | 0.73 (0.67-0.81) | 0.17 (0.15-0.19) | 0.78 (0.76-0.79) |
| XGBoost Preop | 0.75 (0.65-0.81) | 0.68 (0.63-0.77) | 0.12 (0.10-0.14) | 0.98 (0.97-0.98) | 0.68 (0.63-0.76) | 0.21 (0.18-0.23) | 0.78 (0.77-0.79) |
| Deep Preop | 0.74 (0.70-0.83) | 0.76 (0.65-0.79) | 0.15 (0.12-0.16) | 0.98 (0.98-0.99) | 0.76 (0.66-0.79) | 0.22 (0.20-0.24) | 0.82 (0.81-0.83) |
| Deep Preop (Multi-Task) | 0.78 (0.71-0.83) | 0.72 (0.67-0.78) | 0.14 (0.13-0.16) | 0.98 (0.98-0.99) | 0.72 (0.68-0.78) | 0.26 (0.23-0.29) | 0.82 (0.81-0.83) |
| Random Forest Intraop | 0.52 (0.50-0.64) | 0.74 (0.64-0.75) | 0.10 (0.09-0.11) | 0.96 (0.96-0.97) | 0.73 (0.63-0.73) | 0.12 (0.11-0.13) | 0.67 (0.66-0.69) |
| XGBoost Intraop | 0.55 (0.45-0.65) | 0.70 (0.59-0.79) | 0.09 (0.08-0.12) | 0.96 (0.96-0.97) | 0.69 (0.60-0.77) | 0.13 (0.11-0.14) | 0.66 (0.65-0.68) |
| Deep Intraop | 0.59 (0.51-0.65) | 0.75 (0.69-0.82) | 0.12 (0.10-0.14) | 0.97 (0.97-0.97) | 0.74 (0.69-0.80) | 0.16 (0.14-0.18) | 0.71 (0.69-0.73) |
| Deep Intraop (Multi-Task) | 0.62 (0.54-0.71) | 0.73 (0.65-0.81) | 0.12 (0.10-0.15) | 0.97 (0.97-0.98) | 0.73 (0.65-0.80) | 0.17 (0.15-0.19) | 0.74 (0.72-0.75) |
| Random Forest Postop | 0.73 (0.70-0.76) | 0.67 (0.66-0.68) | 0.11 (0.11-0.12) | 0.98 (0.97-0.98) | 0.67 (0.67-0.68) | 0.14 (0.13-0.16) | 0.75 (0.74-0.77) |
| XGBoost Postop | 0.68 (0.64-0.76) | 0.73 (0.67-0.77) | 0.13 (0.11-0.14) | 0.98 (0.97-0.98) | 0.73 (0.68-0.77) | 0.19 (0.18-0.22) | 0.77 (0.76-0.79) |
| Deep Postop | 0.78 (0.70-0.83) | 0.72 (0.68-0.78) | 0.14 (0.12-0.16) | 0.98 (0.98-0.99) | 0.72 (0.69-0.78) | 0.23 (0.21-0.26) | 0.82 (0.81-0.83) |
| Deep Postop (Multi-Task) | 0.80 (0.72-0.85) | 0.70 (0.66-0.78) | 0.13 (0.12-0.16) | 0.98 (0.98-0.99) | 0.70 (0.67-0.78) | 0.25 (0.22-0.27) | 0.83 (0.81-0.84) |
| **In-Hospital Mortality** | | | | | | | |
| Random Forest Preop | 0.75 (0.70-0.80) | 0.82 (0.82-0.83) | 0.06 (0.06-0.07) | 1.00 (0.99-1.00) | 0.82 (0.81-0.83) | 0.12 (0.10-0.15) | 0.84 (0.82-0.86) |
| XGBoost Preop | 0.83 (0.70-0.88) | 0.75 (0.68-0.87) | 0.05 (0.04-0.08) | 1.00 (0.99-1.00) | 0.75 (0.68-0.86) | 0.16 (0.13-0.20) | 0.87 (0.85-0.89) |
| Deep Preop | 0.83 (0.80-0.92) | 0.81 (0.72-0.81) | 0.06 (0.05-0.07) | 1.00 (1.00-1.00) | 0.81 (0.73-0.81) | 0.17 (0.14-0.22) | 0.90 (0.88-0.91) |
| Deep Preop (Multi-Task) | 0.79 (0.76-0.88) | 0.84 (0.72-0.85) | 0.07 (0.05-0.08) | 1.00 (1.00-1.00) | 0.83 (0.72-0.85) | 0.17 (0.13-0.20) | 0.89 (0.88-0.91) |
| Random Forest Intraop | 0.74 (0.64-0.79) | 0.72 (0.72-0.84) | 0.04 (0.04-0.06) | 0.99 (0.99-1.00) | 0.72 (0.72-0.84) | 0.08 (0.06-0.10) | 0.79 (0.77-0.82) |
| XGBoost Intraop | 0.77 (0.73-0.83) | 0.80 (0.75-0.81) | 0.06 (0.05-0.07) | 1.00 (0.99-1.00) | 0.80 (0.75-0.81) | 0.17 (0.14-0.22) | 0.86 (0.85-0.88) |
| Deep Intraop | 0.85 (0.73-0.88) | 0.72 (0.71-0.85) | 0.05 (0.04-0.07) | 1.00 (0.99-1.00) | 0.73 (0.71-0.85) | 0.15 (0.12-0.19) | 0.86 (0.84-0.88) |
| Deep Intraop (Multi-Task) | 0.83 (0.73-0.87) | 0.76 (0.74-0.85) | 0.05 (0.05-0.08) | 1.00 (0.99-1.00) | 0.76 (0.74-0.85) | 0.18 (0.14-0.22) | 0.88 (0.86-0.89) |
| Random Forest Postop | 0.72 (0.62-0.79) | 0.82 (0.81-0.89) | 0.06 (0.05-0.09) | 0.99 (0.99-1.00) | 0.82 (0.81-0.88) | 0.11 (0.09-0.13) | 0.83 (0.81-0.86) |
| XGBoost Postop | 0.87 (0.83-0.92) | 0.83 (0.79-0.85) | 0.07 (0.06-0.09) | 1.00 (1.00-1.00) | 0.83 (0.79-0.85) | 0.20 (0.16-0.25) | 0.91 (0.89-0.92) |
| Deep Postop | 0.91 (0.78-0.94) | 0.73 (0.72-0.85) | 0.05 (0.05-0.08) | 1.00 (1.00-1.00) | 0.73 (0.72-0.85) | 0.19 (0.15-0.24) | 0.90 (0.89-0.92) |
| Deep Postop (Multi-Task) | 0.88 (0.79-0.95) | 0.79 (0.74-0.90) | 0.06 (0.05-0.11) | 1.00 (1.00-1.00) | 0.79 (0.74-0.90) | 0.20 (0.16-0.25) | 0.92 (0.91-0.93) |
|  |  |  |  |  |  |  |  |

PPV: positive predictive value; NPV: negative predictive value; AUPRC: area under the precision-recall curve; AUROC: area under the receiver operating characteristic curve.

**Supplementary Table S6. Summary of input features and description of variable preprocessing.**

| **Variable Name** | **Type** | **Data Source** | **Categories** | **Preprocessing** |
| --- | --- | --- | --- | --- |
| **Demographic Variables** | | | | |
| Age | Continuous | Derived |  | Outlier adjustment ^a^, Feature scaling ^c^ |
| Gender | Binary | Raw | 2 |  |
| Race | Nominal | Raw | 3 | Missing value imputation ^b^, One-hot encoding ^d^ |
| Body mass index | Continuous | Raw |  | Outlier adjustment ^a^, Missing value imputation ^b^, Feature scaling ^c^ |
| Marital status | Nominal | Raw | 3 | One-hot encoding ^d^ |
| Ethnicity | Binary | Raw | 2 | Missing value imputation ^b^ |
| Language | Binary | Raw | 2 | Missing value imputation ^b^ |
| Smoking status | Nominal | Raw | 3 | One-hot encoding ^d^ |
| Insurance | Nominal | Raw | 4 | One-hot encoding ^d^ |
| **Patient Neighborhood Characteristics** | | | | |
| ZIP code | Nominal | Raw | 2108 | Missing value imputation ^b^, Embedding representation ^f^ |
| Rural area | Binary | Derived | 2 | Missing value imputation ^b^ |
| Total population | Continuous | Derived |  | Outlier adjustment ^a^, Missing value imputation ^b^, Feature scaling ^c^ |
| Median income | Continuous | Derived |  | Outlier adjustment ^a^, Missing value imputation ^b^, Feature scaling ^c^ |
| African-American population proportion | Continuous | Derived |  | Outlier adjustment ^a^, Missing value imputation ^b^, Feature scaling ^c^ |
| Hispanic population proportion | Continuous | Derived |  | Outlier adjustment ^a^, Missing value imputation ^b^, Feature scaling ^c^ |
| Poverty rate | Continuous | Derived |  | Outlier adjustment ^a^, Missing value imputation ^b^, Feature scaling ^c^ |
| Distance to hospital | Continuous | Derived |  | Outlier adjustment ^a^, Missing value imputation ^b^, Feature scaling ^c^ |
| **Admission Information** | | | | |
| Month of admission | Nominal | Raw | 12 | Cyclical embedding ^g^, Feature scaling ^c^ |
| Day of admission | Nominal | Raw | 7 | Cyclical embedding ^g^, Feature scaling ^c^ |
| Hour of admission | Nominal | Raw | 24 | Cyclical embedding ^g^, Feature scaling ^c^ |
| Admission source | Binary | Raw | 2 |  |
| Admission type (emergent/elective) | Binary | Derived | 2 |  |
| Admission type (medicine/surgery) | Binary | Derived | 2 |  |
| Night admission | Binary | Derived | 2 |  |
| **Surgical Procedure Information** | | | | |
| Primary procedure | Nominal | Derived | 3232 | Embedding representation ^f^ |
| Attending surgeon | Nominal | Raw | 370 | Embedding representation ^f^ |
| Surgery type | Nominal | Derived | 17 | Embedding representation ^f^ |
| Operating room | Nominal | Raw | 63 | Embedding representation ^f^ |
| Operating room type (trauma) | Binary | Derived | 2 |  |
| Anesthesia type | Binary | Derived | 2 |  |
| Scheduled postoperative location | Binary | Derived | 2 |  |
| Time from admission to surgery | Continuous | Derived |  | Outlier adjustment ^a^, Feature scaling ^c^ |
| Surgeon specialty | Nominal | Derived | 48 | Embedding representation ^f^ |
| **Admission comorbidities** | | | | |
| Myocardial infarction | Binary | Derived | 2 |  |
| Congestive heart failure | Binary | Derived | 2 |  |
| Peripheral vascular disease | Binary | Derived | 2 |  |
| Cerebrovascular disease | Binary | Derived | 2 |  |
| Chronic pulmonary disease | Binary | Derived | 2 |  |
| Metastatic carcinoma | Binary | Derived | 2 |  |
| Cancer | Binary | Derived | 2 |  |
| Liver disease | Binary | Derived | 2 |  |
| Diabetes | Binary | Derived | 2 |  |
| Hypertension | Binary | Derived | 2 |  |
| Hypothyroidism | Binary | Derived | 2 |  |
| Valvular disease | Binary | Derived | 2 |  |
| Coagulopathy | Binary | Derived | 2 |  |
| Obesity | Binary | Derived | 2 |  |
| Weight loss | Binary | Derived | 2 |  |
| Fluid/electrolyte disorders | Binary | Derived | 2 |  |
| Chronic anemia | Binary | Derived | 2 |  |
| Alcohol or drug abuse | Binary | Derived | 2 |  |
| Depression | Binary | Derived | 2 |  |
| Charlson comorbidity index | Continuous | Derived |  |  |
| Number of unique diagnosis codes | Continuous | Derived |  |  |
| Chronic kidney disease | Binary | Derived | 2 |  |
| **Medications History** | | | | |
| ACE Inhibitors | Binary | Derived | 2 |  |
| Aminoglycosides | Binary | Derived | 2 |  |
| Antiemetics | Binary | Derived | 2 |  |
| Aspirin | Binary | Derived | 2 |  |
| Beta Blockers | Binary | Derived | 2 |  |
| Bicarbonates | Binary | Derived | 2 |  |
| Corticosteroids | Binary | Derived | 2 |  |
| Diuretics | Binary | Derived | 2 |  |
| NSAIDS | Binary | Derived | 2 |  |
| Vasopressors/Inotropes | Binary | Derived | 2 |  |
| Statins | Binary | Derived | 2 |  |
| Vancomycin | Binary | Derived | 2 |  |
| Nephrotoxic | Binary | Derived | 2 |  |
| Total number of medications | Continuous | Derived |  |  |
| **Laboratory Results History** | | | | |
| Urea nitrogen/creatinine ratio | Continuous | Raw |  | Outlier adjustment ^a^, Missing value imputation ^b^, Feature scaling ^c^ |
| Hemoglobin | Continuous | Raw |  | Outlier adjustment ^a^, Missing value imputation ^b^, Feature scaling ^c^ |
| Leukocytes | Continuous | Raw |  | Outlier adjustment ^a^, Missing value imputation ^b^, Feature scaling ^c^ |
| Erythrocytes | Continuous | Raw |  | Outlier adjustment ^a^, Missing value imputation ^b^, Feature scaling ^c^ |
| Hematocrit | Continuous | Raw |  | Outlier adjustment ^a^, Missing value imputation ^b^, Feature scaling ^c^ |
| Erythrocyte mean corpuscular volume | Continuous | Raw |  | Outlier adjustment ^a^, Missing value imputation ^b^, Feature scaling ^c^ |
| Erythrocyte mean corpuscular hemoglobin concentration | Continuous | Raw |  | Outlier adjustment ^a^, Missing value imputation ^b^, Feature scaling ^c^ |
| Erythrocyte mean corpuscular hemoglobin | Continuous | Raw |  | Outlier adjustment ^a^, Missing value imputation ^b^, Feature scaling ^c^ |
| Erythrocyte distribution width | Continuous | Raw |  | Outlier adjustment ^a^, Missing value imputation ^b^, Feature scaling ^c^ |
| Platelets | Continuous | Raw |  | Outlier adjustment ^a^, Missing value imputation ^b^, Feature scaling ^c^ |
| Platelet mean volume | Continuous | Raw |  | Outlier adjustment ^a^, Missing value imputation ^b^, Feature scaling ^c^ |
| Neutrophils | Continuous | Raw |  | Outlier adjustment ^a^, Missing value imputation ^b^, Feature scaling ^c^ |
| Glucose, serum | Continuous | Raw |  | Outlier adjustment ^a^, Missing value imputation ^b^, Feature scaling ^c^ |
| Urea nitrogen, serum | Continuous | Raw |  | Outlier adjustment ^a^, Missing value imputation ^b^, Feature scaling ^c^ |
| Creatinine, serum | Continuous | Raw |  | Outlier adjustment ^a^, Missing value imputation ^b^, Feature scaling ^c^ |
| Sodium, serum | Continuous | Raw |  | Outlier adjustment ^a^, Missing value imputation ^b^, Feature scaling ^c^ |
| Potassium, serum | Continuous | Raw |  | Outlier adjustment ^a^, Missing value imputation ^b^, Feature scaling ^c^ |
| Chloride, serum | Continuous | Raw |  | Outlier adjustment ^a^, Missing value imputation ^b^, Feature scaling ^c^ |
| Carbon dioxide, serum | Continuous | Raw |  | Outlier adjustment ^a^, Missing value imputation ^b^, Feature scaling ^c^ |
| Lactate, serum | Continuous | Raw |  | Outlier adjustment ^a^, Missing value imputation ^b^, Feature scaling ^c^ |
| Calcium, serum | Continuous | Raw |  | Outlier adjustment ^a^, Missing value imputation ^b^, Feature scaling ^c^ |
| Anion gap, serum | Continuous | Raw |  | Outlier adjustment ^a^, Missing value imputation ^b^, Feature scaling ^c^ |
| Alanine, serum | Continuous | Raw |  | Outlier adjustment ^a^, Missing value imputation ^b^, Feature scaling ^c^ |
| Albumin, serum | Continuous | Raw |  | Outlier adjustment ^a^, Missing value imputation ^b^, Feature scaling ^c^ |
| Asparate, serum | Continuous | Raw |  | Outlier adjustment ^a^, Missing value imputation ^b^, Feature scaling ^c^ |
| Bilirubin, serum | Continuous | Raw |  | Outlier adjustment ^a^, Missing value imputation ^b^, Feature scaling ^c^ |
| C-reactive protein, serum | Continuous | Raw |  | Outlier adjustment ^a^, Missing value imputation ^b^, Feature scaling ^c^ |
| Prothrombin time, serum | Continuous | Raw |  | Outlier adjustment ^a^, Missing value imputation ^b^, Feature scaling ^c^ |
| Erythrocyte sedimentation rate, serum | Continuous | Raw |  | Outlier adjustment ^a^, Missing value imputation ^b^, Feature scaling ^c^ |
| Troponin I, serum | Continuous | Raw |  | Outlier adjustment ^a^, Missing value imputation ^b^, Feature scaling ^c^ |
| Troponin T, serum | Continuous | Raw |  | Outlier adjustment ^a^, Missing value imputation ^b^, Feature scaling ^c^ |
| Erythrocytes, urine | Nominal | Derived | 4 | Missing value imputation ^b^, One-hot encoding ^d^ |
| Protein, urine | Nominal | Derived | 4 | Missing value imputation ^b^, One-hot encoding ^d^ |
| Glucose, urine | Nominal | Derived | 4 | Missing value imputation ^b^, One-hot encoding ^d^ |
| Hemoglobin, urine | Nominal | Derived | 4 | Missing value imputation ^b^, One-hot encoding ^d^ |
| Reference creatinine | Continuous | Derived |  | Outlier adjustment ^a^, Missing value imputation ^b^, Feature scaling ^c^ |
| Estimated glomerular filtration rate | Continuous | Derived |  | Outlier adjustment ^a^, Missing value imputation ^b^, Feature scaling ^c^ |
| **Intraoperative Variables** | | | | |
| Systolic blood pressure | Continuous | Raw |  | Temporal processing ^i^, Time series creation ^j^, Feature scaling ^c^, Baseline time series extraction ^k^ |
| Diastolic blood pressure | Continuous | Raw |  | Temporal processing ^i^, Time series creation ^j^, Feature scaling ^c^, Baseline time series extraction ^k^ |
| Mean arterial pressure | Continuous | Raw |  | Temporal processing ^i^, Time series creation ^j^, Feature scaling ^c^, Baseline time series extraction ^k^ |
| Heart rate | Continuous | Raw |  | Temporal processing ^i^, Time series creation ^j^, Feature scaling ^c^, Baseline time series extraction ^k^ |
| Oxygen saturation (SpO2) | Continuous | Raw |  | Temporal processing ^i^, Time series creation ^j^, Feature scaling ^c^, Baseline time series extraction ^k^ |
| Fraction of inspired oxygen (FiO2) | Continuous | Raw |  | Temporal processing ^i^, Time series creation ^j^, Feature scaling ^c^, Baseline time series extraction ^k^ |
| End-tidal carbon dioxide (EtCO2) | Continuous | Raw |  | Temporal processing ^i^, Time series creation ^j^, Feature scaling ^c^, Baseline time series extraction ^k^ |
| Tidal volume | Continuous | Raw |  | Temporal processing ^i^, Time series creation ^j^, Feature scaling ^c^, Baseline time series extraction ^k^ |
| Respiration rate | Continuous | Raw |  | Temporal processing ^i^, Time series creation ^j^, Feature scaling ^c^, Baseline time series extraction ^k^ |
| Peak inspiratory pressure | Continuous | Raw |  | Temporal processing ^i^, Time series creation ^j^, Feature scaling ^c^, Baseline time series extraction ^k^ |
| Minimum alveolar concentration | Continuous | Raw |  | Temporal processing ^i^, Time series creation ^j^, Feature scaling ^c^, Baseline time series extraction ^k^ |
| Core temperature | Continuous | Raw |  | Temporal processing ^i^, Time series creation ^j^, Feature scaling ^c^, Baseline time series extraction ^k^ |
| Urine output sum | Continuous | Raw |  | Outlier adjustment ^a^, Missing value imputation ^b^, Feature scaling ^c^ |
| Blood loss sum | Continuous | Raw |  | Outlier adjustment ^a^, Missing value imputation ^b^, Feature scaling ^c^ |
| Surgery duration | Continuous | Raw |  | Outlier adjustment ^a^, Missing value imputation ^b^, Feature scaling ^c^ |
|  |  |  |  |  |
| ^a^ For continuous variables, values that fell in the top and bottom 1% of its distribution were considered outliers and capped to the respective values given at the 1^st^ and 99^th^ percentiles. | | | | |
| ^b^ Missing numerical values were replaced with the median from the development cohort, and missing nominal variables were assigned to a distinct “missing” category. | | | | |
| ^c^ Continuous variables were standardized to zero mean and unit variance. | |  |  |  |
| ^d^ Nominal variables with less than 10 levels were represented as zero vectors of length equal to the number of levels, with level indicators equal to one. | | | | |
| ^e^ Using residency zip code, we linked to US Census data to calculate residing neighborhood characteristics and distance from hospital. | | | | |
| ^f^ Nominal variables with 10 levels or greater were transformed to a numeric integer identifier ranging from 0 to the number of unique levels minus one, where implicit variable representations were learned as part of the model training process. | | | | |
| ^g^ To preserve relative proximity, temporally recurring features such as month and day of admission were cyclically embedded as two separate features by sine and cosine-based transformation. For example, December (12) is near January (1), and Sunday (7) is near Monday (1). | | | | |
| ^h^ Medications were taken within one year timeframe prior to surgery using RxNorms data grouped into drug classes according to the US, Department of Veterans Affairs National Drug File-Reference Terminology ^24^. | | | | |
| ^i^ Measurement values lying outside of expert-defined clinically normal value ranges for each variable were discarded. If two measurements existed at the same timestamp for a given patient, a random measurement was kept. | | | | |
| ^j^ For each surgical procedure, a time series was constructed by arranging intraoperative measurements chronologically, resampling to one-minute frequency intervals, performing linear interpolation in both directions (except for blood loss and urine output, which were imputed with zero), and imputing the development median at every timestep for procedures lacking a single measurement of a particular variable. | | | | |
| ^k^ For baseline models, a set of 49 statistical features was extracted from each intraoperative time series. This set included the following features: minimum, maximum, mean, median, standard deviation, sum of values, variance, kurtosis, skewness, absolute energy, absolute sum of changes, counts above and below mean, first and last locations of both minimum and maximum, sequence length, longest strike above and below mean, mean absolute change, mean change, ratio of unique values to sequence length, variance larger than standard deviation, 9 quantiles, 9 index mass quantiles, 10-binned entropy, number of peaks, and range count. | | | | |

**Supplementary Table S7. Net reclassification index results.**

|  |  |  |  |  |  |  |
| --- | --- | --- | --- | --- | --- | --- |
| **Complication** | **Model** | **NRI** | **p** | **Event** | **Non-Event** | **Overall** |
| Prolonged ICU Stay | Random Forest | 0.06 (0.04-0.07) | <0.01 | -0.49 | 6.11 | 3.91 |
|  | XGBoost | 0.07 (0.06-0.08) | <0.01 | 2.68 | 4.51 | 3.90 |
|  | Deep Learning | 0.07 (0.06-0.08) | <0.01 | 3.49 | 3.67 | 3.61 |
|  | Deep Learning (Multi-Task) | 0.07 (0.06-0.08) | <0.01 | 2.25 | 4.48 | 3.74 |
| Prolonged Mechanical Ventilation | Random Forest | 0.12 (0.09-0.14) | <0.01 | 5.02 | 6.74 | 6.61 |
|  | XGBoost | 0.10 (0.08-0.13) | <0.01 | 8.26 | 2.17 | 2.64 |
|  | Deep Learning | 0.07 (0.05-0.09) | <0.01 | 7.31 | -0.25 | 0.34 |
|  | Deep Learning (Multi-Task) | 0.08 (0.06-0.10) | <0.01 | 2.92 | 4.97 | 4.81 |
| Wound Complications | Random Forest | -0.02 (-0.04--0.00) | 0.05 | -1.77 | -0.36 | -0.66 |
|  | XGBoost | -0.01 (-0.02-0.01) | 0.35 | -5.94 | 4.97 | 2.64 |
|  | Deep Learning | 0.03 (0.01-0.04) | <0.01 | 6.79 | -4.03 | -1.71 |
|  | Deep Learning (Multi-Task) | 0.02 (0.00-0.03) | 0.07 | 7.25 | -5.67 | -2.90 |
| Neurological Complications | Random Forest | -0.03 (-0.04--0.01) | 0.01 | -3.09 | 0.49 | -0.23 |
|  | XGBoost | -0.01 (-0.02-0.01) | 0.57 | -0.12 | -0.38 | -0.33 |
|  | Deep Learning | 0.01 (-0.00-0.02) | 0.21 | 3.04 | -2.21 | -1.14 |
|  | Deep Learning (Multi-Task) | 0.01 (-0.00-0.02) | 0.44 | 3.26 | -2.73 | -1.52 |
| Cardiovascular Complications | Random Forest | 0.08 (0.06-0.09) | <0.01 | -0.27 | 7.90 | 6.57 |
|  | XGBoost | 0.08 (0.07-0.10) | <0.01 | 3.54 | 4.94 | 4.71 |
|  | Deep Learning | 0.07 (0.06-0.09) | <0.01 | 5.03 | 2.40 | 2.83 |
|  | Deep Learning (Multi-Task) | 0.09 (0.08-0.11) | <0.01 | 10.78 | -1.75 | 0.29 |
| Sepsis | Random Forest | -0.00 (-0.02-0.02) | 0.86 | 1.63 | -1.84 | -1.54 |
|  | XGBoost | 0.02 (-0.00-0.04) | 0.23 | 1.80 | -0.21 | -0.03 |
|  | Deep Learning | 0.02 (0.00-0.03) | 0.07 | 0.85 | 0.95 | 0.94 |
|  | Deep Learning (Multi-Task) | 0.01 (-0.00-0.03) | 0.17 | -1.97 | 3.31 | 2.85 |
| Acute Kidney Injury | Random Forest | 0.01 (-0.00-0.03) | 0.22 | -1.69 | 3.01 | 2.21 |
|  | XGBoost | -0.00 (-0.02-0.01) | 0.71 | 1.80 | -2.18 | -1.51 |
|  | Deep Learning | 0.01 (-0.00-0.02) | 0.25 | -4.62 | 5.51 | 3.79 |
|  | Deep Learning (Multi-Task) | 0.01 (-0.00-0.02) | 0.36 | -0.38 | 1.06 | 0.82 |
| Venous Thromboembolism | Random Forest | -0.02 (-0.05-0.01) | 0.31 | 4.00 | -6.11 | -5.56 |
|  | XGBoost | -0.01 (-0.04-0.02) | 0.63 | -6.45 | 5.41 | 4.77 |
|  | Deep Learning | 0.00 (-0.02-0.02) | 0.94 | 3.81 | -3.71 | -3.30 |
|  | Deep Learning (Multi-Task) | 0.01 (-0.01-0.03) | 0.68 | 2.63 | -2.06 | -1.81 |
| In-Hospital Mortality | Random Forest | -0.03 (-0.09-0.02) | 0.38 | -3.12 | -0.25 | -0.29 |
|  | XGBoost | 0.12 (0.08-0.17) | <0.01 | 4.05 | 7.94 | 7.87 |
|  | Deep Learning | 0.00 (-0.04-0.04) | 0.92 | 8.41 | -8.13 | -7.87 |
|  | Deep Learning (Multi-Task) | 0.05 (0.01-0.08) | 0.07 | 9.35 | -4.57 | -4.35 |
|  |  |  |  |  |  |  |

**Supplementary Table S8. Absolute and relative risk between preoperative and postoperative models.**

| **Outcome** | **Prevalence (%)** | **Preoperative** | | | | | **Postoperative** | | | | |
| --- | --- | --- | --- | --- | --- | --- | --- | --- | --- | --- | --- |
|  |  | **Low Risk** | | **High Risk** | | **Relative Risk (%)** | **Low Risk** | | **High Risk** | | **Relative Risk (%) (+/- Preoperative)** |
|  |  | **N (%)** | **Absolute Risk (%)** | **N (%)** | **Absolute Risk (%)** |  | **N (%)** | **Absolute Risk (+/- Preoperative)** | **N (%)** | **Absolute Risk (+/- Preoperative)** |  |
| Prolonged ICU Stay | 33.3 | 11899 (58.6) | 10.9 | 8394 (41.4) | 65.2 | 6.0 | 12353 (60.9) | 9.2 (-1.6) | 7940 (39.1) | 70.8 (+5.6) | 7.7 (+1.7) |
| Prolonged Mechanical Ventilation | 7.8 | 15140 (74.6) | 1.7 | 5153 (25.4) | 25.6 | 15.1 | 16025 (79.0) | 1.3 (-0.4) | 4268 (21.0) | 32.0 (+6.4) | 24.4 (+9.3) |
| Wound Complications | 21.4 | 12932 (63.7) | 11.2 | 7361 (36.3) | 39.5 | 3.5 | 11713 (57.7) | 9.6 (-1.5) | 8580 (42.3) | 37.5 (-1.9) | 3.9 (+0.4) |
| Neurological Complications | 20.2 | 12808 (63.1) | 6.6 | 7485 (36.9) | 43.6 | 6.6 | 12232 (60.3) | 5.8 (-0.8) | 8061 (39.7) | 42.2 (-1.5) | 7.3 (+0.6) |
| Cardiovasc.  Complications | 16.3 | 13505 (66.6) | 6.9 | 6788 (33.4) | 34.9 | 5.1 | 12851 (63.3) | 4.5 (-2.4) | 7442 (36.7) | 36.6 (+1.7) | 8.2 (+3.1) |
| Sepsis | 8.7 | 14823 (73.0) | 2.4 | 5470 (27.0) | 25.9 | 10.7 | 15471 (76.2) | 2.6 (+0.1) | 4822 (23.8) | 28.6 (+2.8) | 11.2 (+0.6) |
| Acute Kidney Injury | 16.9 | 13442 (66.2) | 6.6 | 6851 (33.8) | 37.2 | 5.6 | 13634 (67.2) | 6.6 (0.0) | 6659 (32.8) | 38.1 (+0.9) | 5.7 (+0.1) |
| Venous Thromb. | 5.4 | 14029 (69.1) | 1.7 | 6264 (30.9) | 13.7 | 7.9 | 13604 (67.0) | 1.6 (-0.2) | 6689 (33.0) | 13.2 (-0.4) | 8.4 (+0.5) |
| In-Hospital Mortality | 1.6 | 16754 (82.6) | 0.4 | 3539 (17.4) | 7.1 | 17.6 | 15812 (77.9) | 0.2 (-0.2) | 4481 (22.1) | 6.3 (-0.8) | 26.3 (+8.7) |

**Supplementary Table S9. Risk group transitions and corresponding inpatient mortality rate.**

| **Outcome** | **Preoperative Risk Group** | **Postoperative Risk Group** | **N** | **% Encounters** | **% Preoperative Risk Group** | **Transition Group Mortality Rate** |
| --- | --- | --- | --- | --- | --- | --- |
| **Prolonged ICU Stay** | High | High | 6705 | 33.0 | 79.9 | 4.4 |
|  | High | Low | 1689 | 8.3 | 20.1 | 0.5 |
|  | Low | High | 1235 | 6.1 | 10.4 | 0.6 |
|  | Low | Low | 10664 | 52.6 | 89.6 | 0.1 |
| **Prolonged Mechanical Ventilation** | High | High | 3514 | 17.3 | 68.2 | 7.3 |
|  | High | Low | 1639 | 8.1 | 31.8 | 0.9 |
|  | Low | High | 754 | 3.7 | 5.0 | 2.0 |
|  | Low | Low | 14386 | 70.9 | 95.0 | 0.3 |
| **Wound Complications** | High | High | 6381 | 31.4 | 86.7 | 3.5 |
|  | High | Low | 980 | 4.8 | 13.3 | 0.8 |
|  | Low | High | 2199 | 10.8 | 17.0 | 1.5 |
|  | Low | Low | 10733 | 52.9 | 83.0 | 0.5 |
| **Neurological Complications** | High | High | 6774 | 33.4 | 90.5 | 4.3 |
|  | High | Low | 711 | 3.5 | 9.5 | 0.1 |
|  | Low | High | 1287 | 6.3 | 10.0 | 1.4 |
|  | Low | Low | 11521 | 56.8 | 90.0 | 0.1 |
| **Cardiovascular Complications** | High | High | 5381 | 26.5 | 79.3 | 5.2 |
|  | High | Low | 1407 | 6.9 | 20.7 | 0.6 |
|  | Low | High | 2061 | 10.2 | 15.3 | 0.5 |
|  | Low | Low | 11444 | 56.4 | 84.7 | 0.2 |
| **Sepsis** | High | High | 4269 | 21.0 | 78.0 | 5.5 |
|  | High | Low | 1201 | 5.9 | 22.0 | 1.6 |
|  | Low | High | 553 | 2.7 | 3.7 | 2.5 |
|  | Low | Low | 14270 | 70.3 | 96.3 | 0.4 |
| **Acute Kidney Injury** | High | High | 5792 | 28.5 | 84.5 | 4.6 |
|  | High | Low | 1059 | 5.2 | 15.5 | 0.8 |
|  | Low | High | 867 | 4.3 | 6.4 | 1.6 |
|  | Low | Low | 12575 | 62.0 | 93.6 | 0.2 |
| **Venous Thromboembolism** | High | High | 5431 | 26.8 | 86.7 | 5.0 |
|  | High | Low | 833 | 4.1 | 13.3 | 1.0 |
|  | Low | High | 1258 | 6.2 | 9.0 | 1.2 |
|  | Low | Low | 12771 | 62.9 | 91.0 | 0.2 |
| **In-Hospital Mortality** | High | High | 3041 | 15.0 | 85.9 | 8.2 |
|  | High | Low | 498 | 2.5 | 14.1 | 1.0 |
|  | Low | High | 1440 | 7.1 | 8.6 | 2.4 |
|  | Low | Low | 15314 | 75.5 | 91.4 | 0.2 |

**Supplementary Table S10. Risk transitions categorized by multi-task complication group.**

| **Preoperative** | | **Postoperative** | | **Risk Category Transition (Preoperative to Postoperative)** | | | | | |
| --- | --- | --- | --- | --- | --- | --- | --- | --- | --- |
| **Risk Category** | **N (%)** | **Risk Category** | **N (%)** | **Preoperative Risk Category** | **Postoperative Risk Category** | **N** | **% Encounters** | **% Preoperative Risk Category** | **Transition Group Mortality (%)** |
| All High | 2275 (11.2) | All High | 2397 (11.8) | All High | All High | 1709 | 8.4 | 75.1 | 10.6 |
|  |  |  |  | All High | Mixed | 566 | 2.8 | 24.9 | 2.1 |
|  |  |  |  | All High | All Low | 0 | 0.0 | 0.0 | 0.0 |
| All Low | 6748 (33.3) | All Low | 6808 (33.5) | All Low | All High | 1 | 0.0 | 0.0 | 0.0 |
|  |  |  |  | All Low | Mixed | 1218 | 6.0 | 18.0 | 0.2 |
|  |  |  |  | All Low | All Low | 5529 | 27.2 | 81.9 | 0.0 |
| Mixed | 11270 (55.5) | Mixed | 11088 (54.6) | Mixed | All High | 687 | 3.4 | 6.1 | 4.9 |
|  |  |  |  | Mixed | Mixed | 9304 | 45.8 | 82.6 | 0.9 |
|  |  |  |  | Mixed | All Low | 1279 | 6.3 | 11.3 | 0.1 |

**SUPPLEMENTARY FIGURES**

**Supplementary Figure S1. Ten features with largest integrated gradients attributions across validation cohort.**

**Supplementary Figure S2. Distribution of integrated gradients feature attributions in validation cohort grouped by preoperative and intraoperative feature types.**

**Supplementary Figure S3. Distribution of integrated gradients feature attributions in validation cohort grouped by input variable type.**

**Supplementary Figure S4. Temporal integrated gradients attributions for an example patient developing postoperative cardiovascular complications. The model correctly predicted elevated risk based on intraoperative physiological time series.**

**Supplementary Figure S5. Overview of cohort selection criteria and derivation of development and validation cohort.**

**SUPPLEMENTARY REFERENCES**

**1.** Charlson ME, Pompei P, Ales KL, MacKenzie CR. A new method of classifying prognostic comorbidity in longitudinal studies: development and validation. *J Chronic Dis.* 1987;40(5):373-383.

**2.** Haley RW, Culver DH, Morgan WM, White JW, Emori TG, Hooton TM. Identifying patients at high risk of surgical wound infection. A simple multivariate index of patient susceptibility and wound contamination. *Am J Epidemiol.* Feb 1985;121(2):206-215.

**3.** Culver DH, Horan TC, Gaynes RP, et al. Surgical wound infection rates by wound class, operative procedure, and patient risk index. National Nosocomial Infections Surveillance System. *Am J Med.* Sep 1991;91(3B):152S-157S.

**4.** Lim GB. Acute coronary syndromes: Supplemental oxygen in myocardial infarction. *Nat Rev Cardiol.* Nov 2017;14(11):632.

**5.** Suffredini AF, Fromm RE, Parker MM, et al. The cardiovascular response of normal humans to the administration of endotoxin. *N Engl J Med.* Aug 1989;321(5):280-287.

**6.** de Castilho FM, Ribeiro ALP, Nobre V, Barros G, de Sousa MR. Heart rate variability as predictor of mortality in sepsis: A systematic review. *PLoS One.* 2018;13(9):e0203487.

**7.** Wolberg AS, Rosendaal FR, Weitz JI, et al. Venous thrombosis. *Nat Rev Dis Primers.* 05 2015;1:15006.
